# Supplementary material for: A closed-loop auditory stimulation approach selectively modulates alpha oscillations and sleep onset dynamics in humans
Source: PLoS Biol. 2024 Jun 18;22(6):e3002651. doi: 10.1371/journal.pbio.3002651 (PMC11185466; doi:10.1371/journal.pbio.3002651)
Supplement: S1 File — The resultant was calculated in electrode Fz of the hd-EEG system in experiment 1 using 2 referencing schemes, Laplacian (as used for all analysis in the main manuscript) and right mastoid (to match the referencing scheme used in the αCLAS EEG system). Channel Fz in the hd-EEG system was adjacent to this channel in the αCLAS EEG system, and the mastoid channel in the hd-EEG system was taken from TP10. The resultant values using the right mastoid reference are higher and more similar to those reported for the αCLAS EEG system. Violin plots show the resultant for each targeted phase in experiment 1 in each reference scheme. Black lines represent each participant. Stats indicate output of paired samples t test between the resultant for each reference scheme, *** indicates p < 0.001. Table A. Data from closed-loop EEG device. Table B. Data from high-density EEG device. Fig B. Phase-locking accuracy across conditions—topography of phase-locking accuracy (average resultant) across 4 conditions for experiments 1 and 2, targeting Fz and Pz electrodes, respectively (blue circles). White marks indicate channels at which resultant >0.4 and p < 0.05. Black marks indicate channels at which resultant <0.4 and p < 0.05; p-values from FDR-corrected z-test for non-uniformity. Fig C. Power change ANOVA for experiment 1 –(A) Topography of permutation ANOVA stats for frequencies across the spectrum. Each topoplot is computed using +/- 0.2 Hz around the labelled frequency. Colours and colourbars indicate F statistics. White marks indicate cluster-corrected p-values <0.05. Fig D. Power change ANOVA for experiment 2 –(A) Topography of permutation ANOVA stats for frequencies across the spectrum. Each topoplot is computed using +/- 0.2 Hz around the labelled frequency. Colours and colourbars indicate F statistics. White marks indicate cluster-corrected p-values <0.05. Fig E. Power change ANOVA for experiment 1 –(A) Topography of permutation ANOVA stats for various frequencies in and around [file pbio.3002651.s001.docx]

**Supplementary Information**


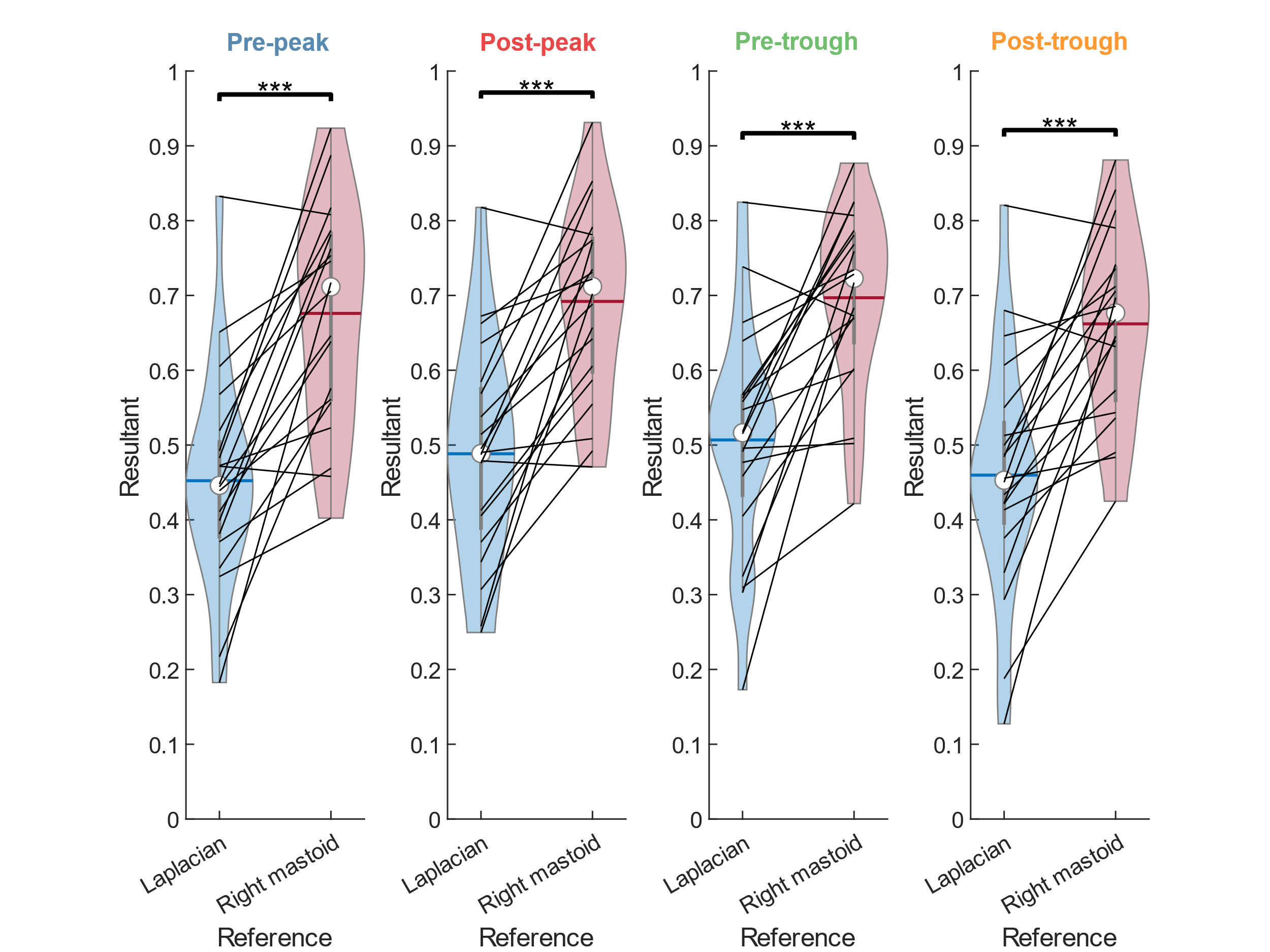


**Figure A. Comparison of the resultant values using different referencing schemes.** The resultant was calculated in electrode Fz of the hd-EEG system in experiment 1 using two referencing schemes, Laplacian (as used for all analysis in the main manuscript) and right mastoid (to match the referencing scheme used in the αCLAS EEG system). Channel Fz in the hd-EEG system was adjacent to this channel in the αCLAS EEG system, and the mastoid channel in the hd-EEG system was taken from TP10. The resultant values using the right mastoid reference are higher and more similar to those reported for the αCLAS EEG system. Violin plots show the resultant for each targeted phase in experiment 1 in each reference scheme. Black lines represent each participant. Stats indicate output of paired samples t-test between the resultant for each reference scheme, *** indicates p < .001

**Tables A and B | Phase-locking accuracy**

Mean phase (ɸ_mean_) and standard deviation (ɸ_SD_) at stimulus onset, and target accuracy for each phase and location targeted in Study 1. The accuracy of phase locking was estimated using the Rayleigh test on the EEG data. Shown are the resultants (R), along with the z-stat (Z) and p values (p) from the Rayleigh test. **S1.1** – Data from the closed-loop EEG device. **S1.2** – Data from the high-density EEG. Note that the two EEG systems are independent and different reference schemes were employed for the closed-loop and high-density EEG data. N indicates number of participants.

| **Table A - Data from closed-loop EEG device** | | | | |
| --- | --- | --- | --- | --- |
|  | **Phase** | | | |
| **Location** | **Pre-Peak 330**° | **Post-Peak 60**° | **Pre-Trough 150**° | **Post-Trough 240**° |
| **Fz (N=23)** | ɸ_mean_ = 327.95°  ɸ_SD_ = 5.38°  R_mean_ = 0.857  R_SD_ = 0.052  Z = 22.88  p = 7.49 × 10^-12^ | ɸ_mean_ = 55.39°  ɸ_SD_ = 5.50°  R_mean_ = 0.861  R_SD_ = .047  Z = 22.82  p = 8.53 × 10^-12^ | ɸ_mean_ = 145.96°  ɸ_SD_ = 7.32°  R_mean_ = 0.847  R_SD_ = 0.044  Z = 22.76  p = 9.72 × 10^-12^ | ɸ_mean_ = 237.99°  ɸ_SD_ = 7.85°  R_mean_ = 0.838  R_SD_ = 0.052  Z = 22.77  p = 9.43 × 10^-12^ |
| **Pz (N=28)** | ɸ_mean_ = 325.23°  ɸ_SD_ = 7.60°  R_mean_ = 0.887  R_SD_ = 0.041  Z = 27.66  p = 7.24 × 10^-14^ | ɸ_mean_ = 53.92°  ɸ_SD_ = 6.14°  R_mean_ = 0.870  R_SD_ = 0.040  Z = 27.68  p = 6.88 × 10^-14^ | ɸ_mean_ = 146.75°  ɸ_SD_ = 9.02°  R_mean_ = 0.870  R_SD_ = 0.045  Z = 27.61  p = 7.99 × 10^-14^ | ɸ_mean_ = 236.30°  ɸ_SD_ = 9.07°  R_mean_ = 0.874  R_SD_ = 0.043  Z = 27.59  p = 8.26 × 10^-14^ |

| **Table B - Data from high-density EEG device** | | | | |
| --- | --- | --- | --- | --- |
|  | **Phase** | | | |
| **Location** | **Pre-Peak 330**° | **Post-Peak 60**° | **Pre-Trough 150**° | **Post-Trough 240**° |
| **Fz (N=23)** | ɸ_mean_ = 353.32°  ɸ_SD_ = 17.75°  R_mean_ = 0.446  R_SD_ = 0.122  Z = 20.26  p = 1.16 × 10^-9^ | ɸ_mean_ = 83.90°  ɸ_SD_ = 17.58°  R _mean_ = 0.473  R_SD_ = 0.118  Z = 20.04  p = 1.73 × 10^--9^ | ɸ_mean_ = 167.87°  ɸ_SD_ = 19.21°  R _mean_ = 0.499  R_SD_ = 0.141  Z = 20.66  p = 5.56 × 10^-10^ | ɸ_mean_ = 256.55°  ɸ_SD_ = 19.04°  R _mean_ = 0.465  R_SD_ = 0.122  Z = 20.83  p = 4.07 × 10^-10^ |
| **Pz (N=28)** | ɸ_mean_ = 343.90°  ɸ_SD_ = 15.77°  R_mean_ = 0.638  R_SD_ = 0.142  Z = 26.15  p = 1.38 × 10^-12^ | ɸ_mean_ = 73.80°  ɸ_SD_ = 13.94  R_mean_ = 0.613  R_SD_ = 0.124  Z = 26.39  p = 8.81 × 10^-13^ | ɸ_mean_ = 161.72°  ɸ_SD_ = 14.50°  R_mean_ = 0.634  R_SD_ = 0.132  Z = 26.54  p = 6.58 × 10^-13^ | ɸ_mean_ = 250.95°  ɸ_SD_ = 14.27°  R_mean_ = 0.622  R_SD_ = 0.129  Z = 26.64  p = 5.41 × 10^-13^ |


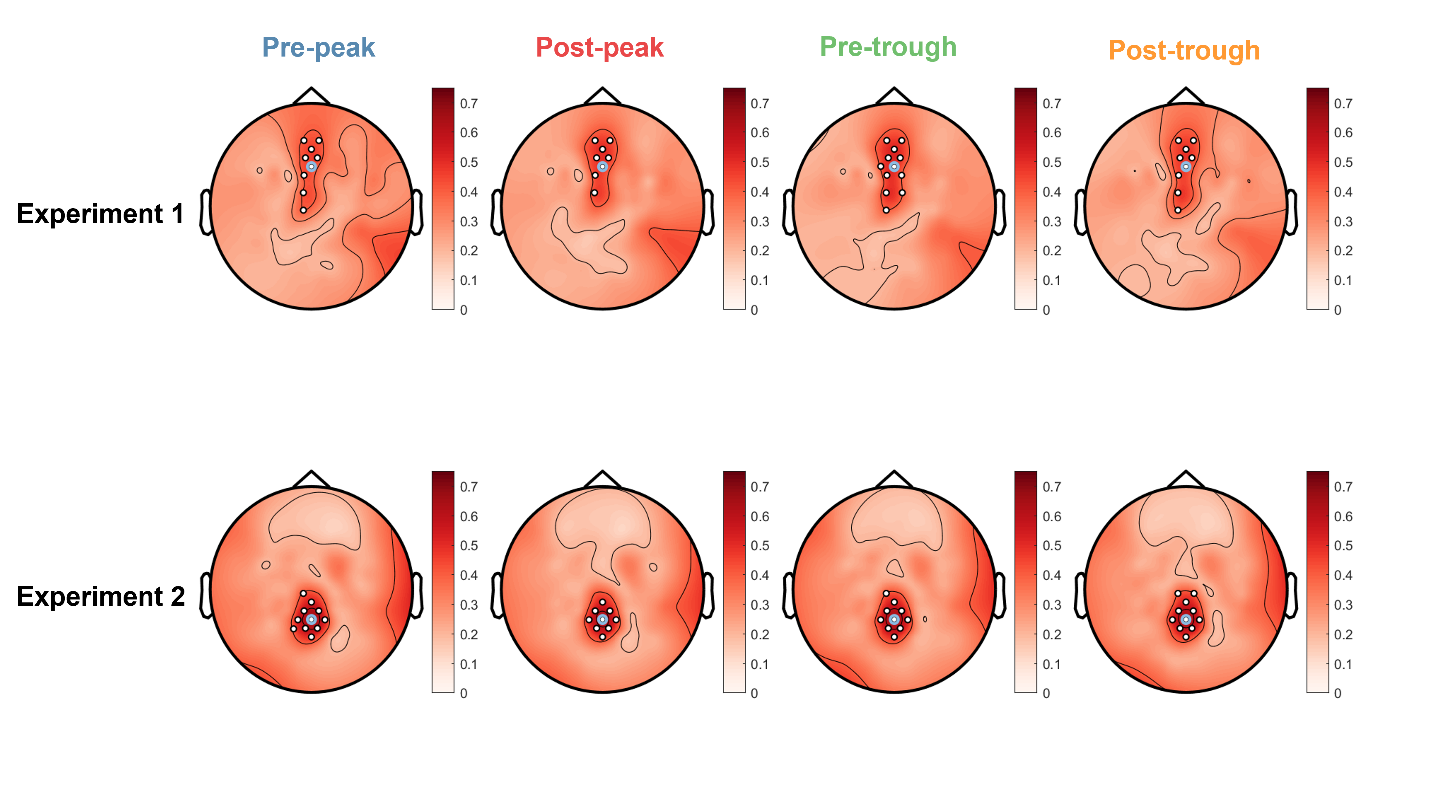


**Figure B. Phase-locking Accuracy Across Conditions** – topography of phase-locking accuracy (average resultant) across 4 conditions for experiments 1 and 2, targeting Fz and Pz electrodes respectively (blue circles). White marks indicate channels at which resultant >0.4 and p <0.05. Black marks indicate channels at which resultant <0.4 and p <0.05. p values from FDR-corrected z-test for non-uniformity.


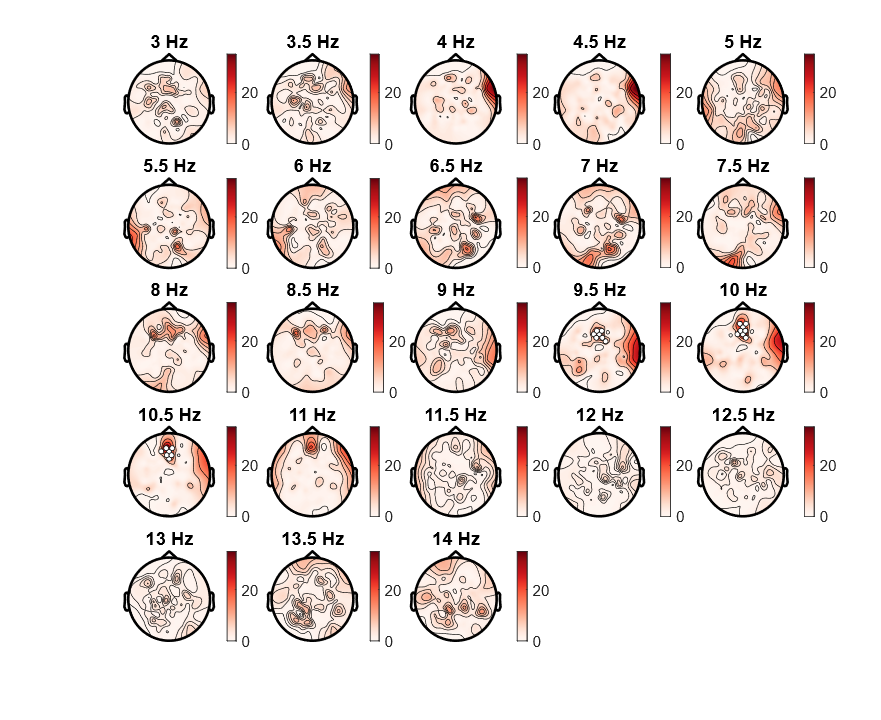
**Figures C and D | power change ANOVA across frequency spectrum**

**Figure C. Power change ANOVA for experiment 1** – (**A)** topography of permutation ANOVA stats for frequencies across the spectrum. Each topoplot is computed using +/- 0.2 Hz around the labelled frequency. Colours and colourbars indicate F statistics. White marks indicate cluster-corrected p-values < .05


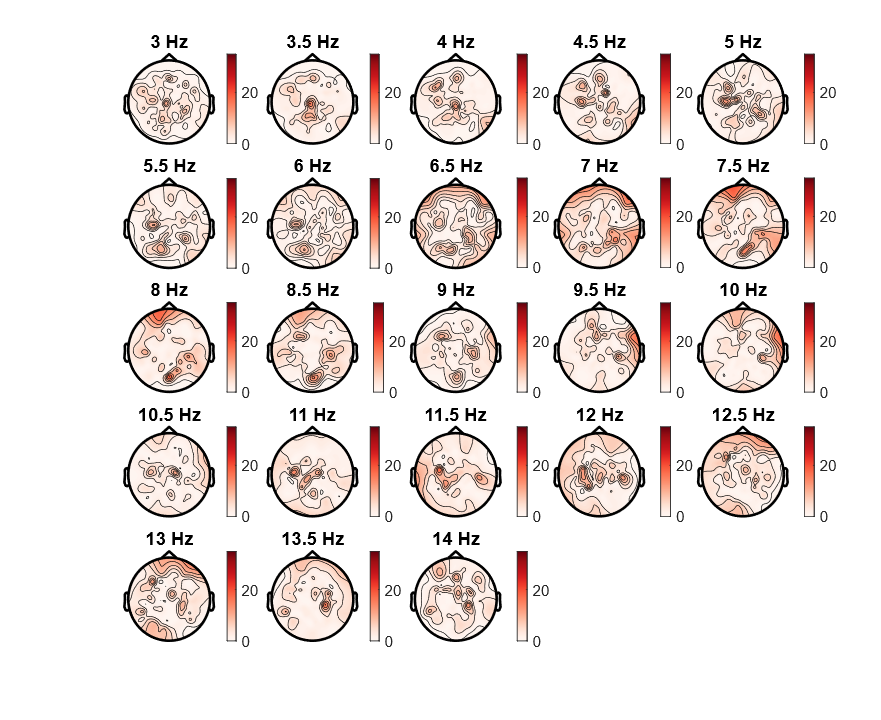
**Figure D. Power change ANOVA for experiment 2** – (**A)** topography of permutation ANOVA stats for frequencies across the spectrum. Each topoplot is computed using +/- .2 Hz around the labelled frequency. Colours and colourbars indicate F statistics. White marks indicate cluster-corrected p-values < .05

**Figures E and F | power change within alpha band**


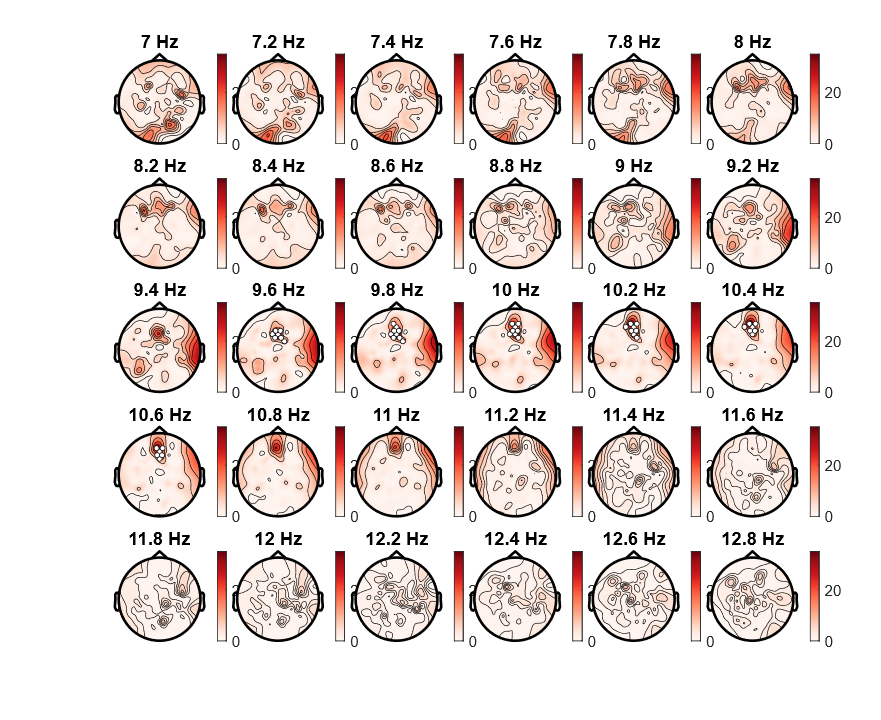


**Figure E. Power change ANOVA for experiment 1** – (**A)** topography of permutation ANOVA stats for various frequencies in and around the alpha band. Each topoplot is computed using +/- 0.2 Hz around the labelled frequency. Colours and colourbars indicate F statistics. White marks indicate cluster-corrected p-values < .05


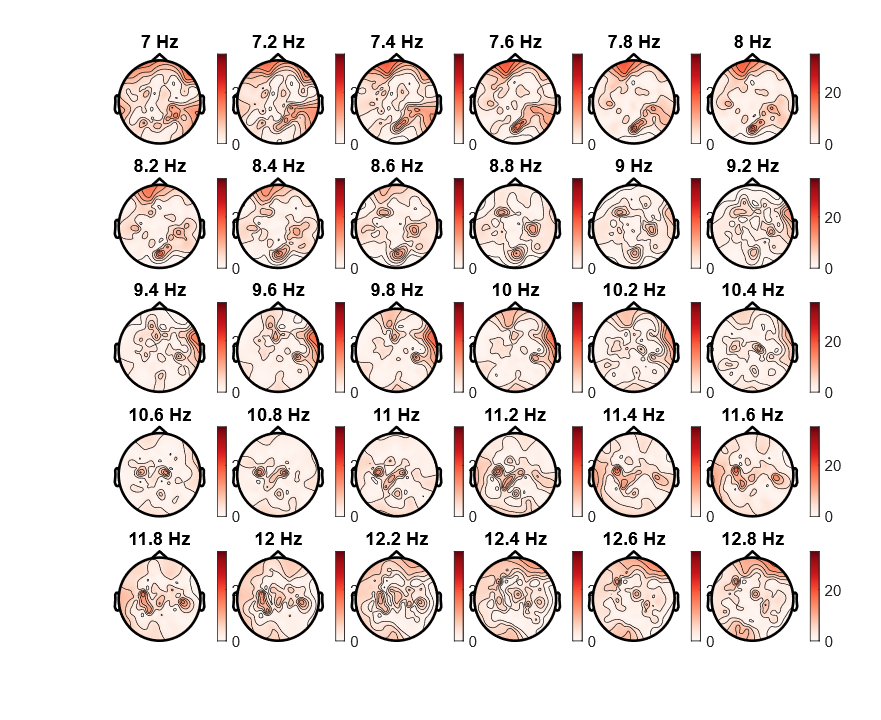
**Figure F. Power change ANOVA for experiment 2** – (**A)** topography of permutation ANOVA stats for various frequencies in and around the alpha band. Each topoplot is computed using +/- 0.2 Hz around the labelled frequency. Colours and colourbars indicate F statistics. White marks indicate cluster-corrected p-values < .05


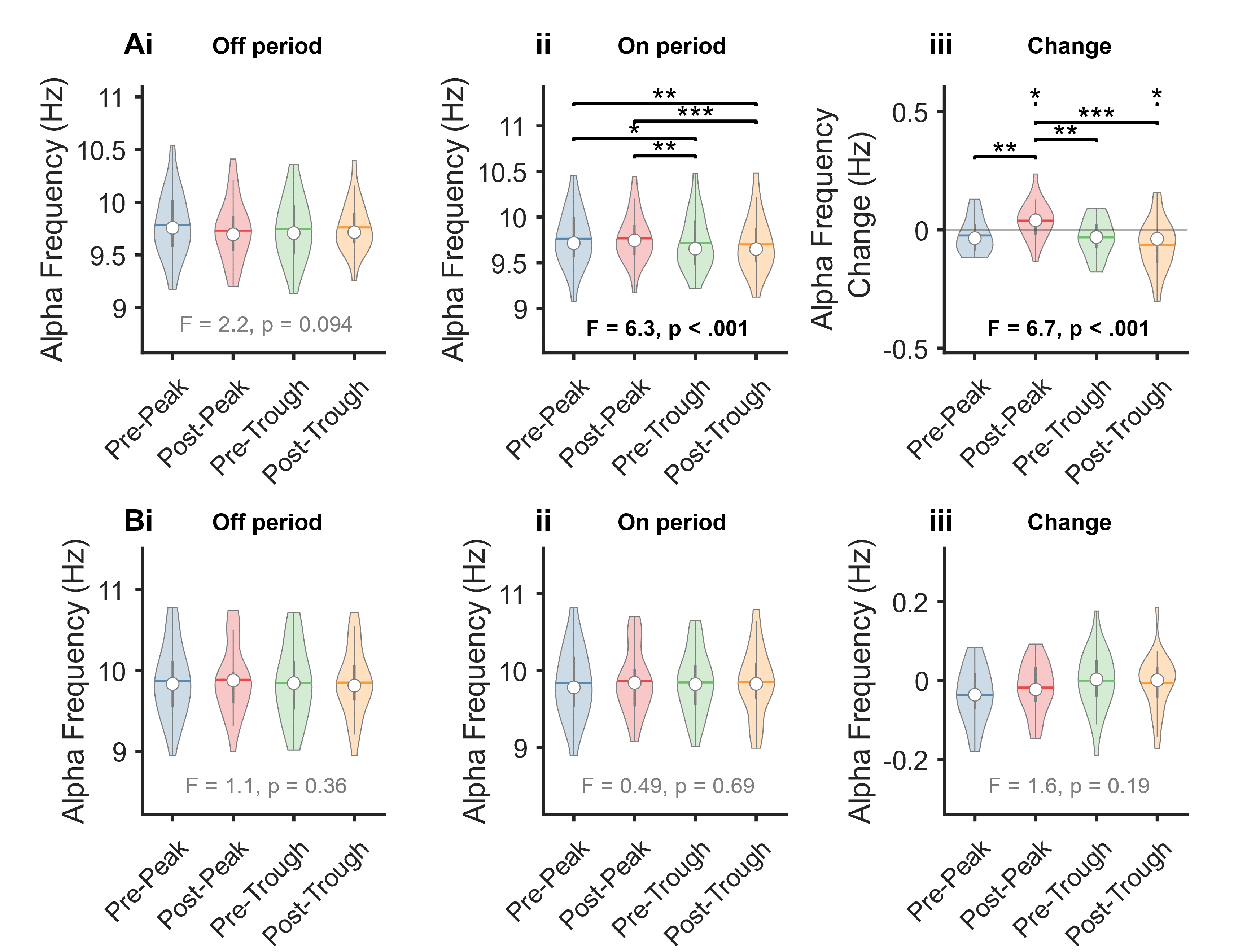


**Figure G. Frequency estimates from off and on periods in experiments 1 and 2**. **(A)** frequency at frontal ROI across four conditions in experiment 1 for **(i)** the ‘off’ period, **(ii)** the ‘on’ period, and **(iii)** the off period subtracted from the on period. **(B)** the same plots but for the parietal ROI in experiment 2. For all plots, mixed effects models were run: [frequency ~ condition + (1|Participant)]. Post-hoc Wald tests were run for those which showed a statistically significant effect of condition (*p* < 0.05). * *p*<0.05, ***p*<0.01, ****p*<.001. Bars between conditions indicate differences between conditions, asterisks above conditions indicate a significant difference from zero, as per one-sample t-test.


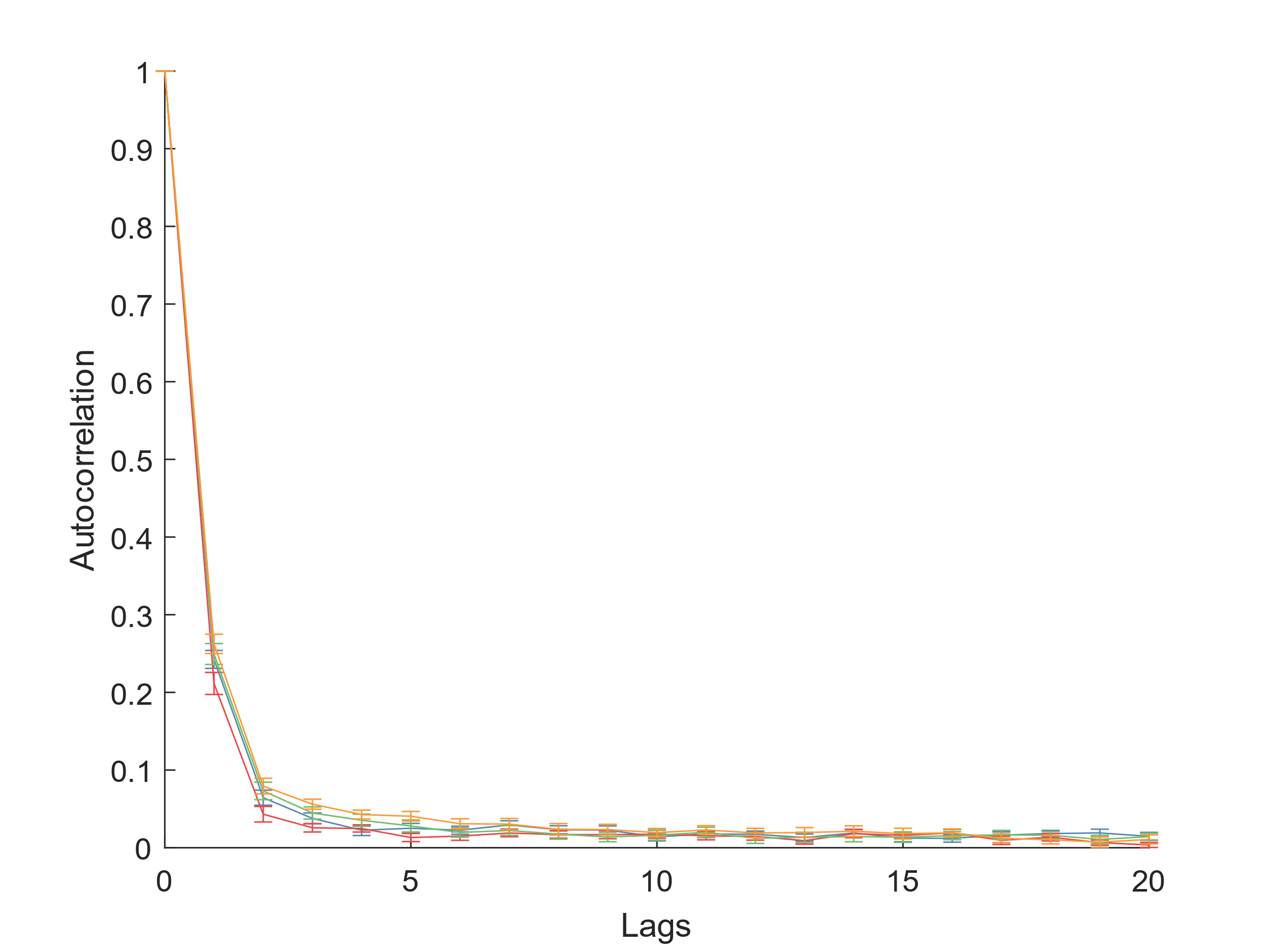


**Figure H Autocorrelation of the inter stimulus interval in experiment 1.** Shown is the autocorrelation coefficient of inter-stimulus intervals across 20 lags, in which a value of 1 would indicate a perfectly periodic stimulus and lower values indicate a deviation from periodicity. The 4 different colours represent the four phase conditions and the errorbars indicate standard error of the mean. It is clear that the stimulation we administered is far from periodic, with the autocorrelation coefficient quickly dropping to <0.1 after only 2 lags. We propose that if the sound was simply entraining itself, via the EEG, then we would see a highly periodic stimulus. This is not the case here.

**Supplementary analysis on αCLAS changes in connectivity**

We evaluated whether αCLAS altered brain connectivity in a phase-dependent manner by using two well-established and complementary, phase-based measures of synchrony, the phase-locking value (PLV) and phase lag index (PLI). While PLV is more sensitive to type 1 errors, i.e. incorrectly assigning connectivity (rejecting the null) that is in fact volume conduction, PLI is more sensitive to type 2 errors, i.e. incorrectly rejecting genuine connectivity (failing to reject the null) with zero (modulo pi) phase difference.

We observed a significant effect of αCLAS phase on the alpha phase-locking value (PLV) connectivity of electrodes around the phase-locking site and the reference used in the αCLAS EEG system (right mastoid) when targeting the alpha rhythm at Fz, experiment 1 (**Figure** **I Ai**), which was specific to the alpha band (**Figure** **J**). To better understand the effect of αCLAS phase on connectivity we computed PLV for each channel pair, per phase condition (**Figure I Bi**). The most noticeable patterns were observed for the post-peak and post-through conditions, such that during post-peak stimulation there was an increase in connectivity, particularly between frontal midline and right temporal channels. The post-through condition, on the other hand, resulted in a fairly global decrease in connectivity, which was also present, albeit to a smaller extent, for the pre-peak and pre-through conditions.

When averaging across all connections with the a priori defined frontal ROI (see Methods), and across time (**Figure I Ci**), a similar pattern is observed to that of alpha frequency change (**Figure 2 Dii**, in main manuscript). We also observed the effect of target phase on PLV was fairly consistent across time (**Figure I Di**).

Using PLI, we observed similar effects of phase on connectivity at the right mastoid, but the previously described frontal midline cluster was not present (**Figure I Aii**), suggesting that the effect here might be due to zero-lag phase-differences. However, when analysing all channel pairs, comparable patterns to PLV of local and long-range connectivity between the frontal midline cluster and right temporal channels were observed (**Figure I Bii**). Regarding PLI per phase condition, while the overall pattern was similar to PLV, long-range connectivity was less salient, suggesting either the presence of volume conduction or the true existence of zero modulo pi-lag connectivity between these regions (**Figure I Bii**). The effect was also clearly less strong when viewed collapsed across the frontal ROI (**Figure I Cii**) and when viewed over time (**Figure I Dii**), with the main differences existing in the post-trough condition.

Analysis of connectivity for experiment 2 (i.e. targeting Pz), did not show an effect of phase on either alpha PLV (**Figure I Ei**) or alpha PLI (**Figure I Eii**), although a small and disparate effect was seen for delta PLV (**Figure** **K**). Exploratory analysis on the alpha connectivity patterns of each phase condition showed a generalised decrease in whole-brain connectivity, particularly for pre-through and post-through conditions, in both PLV (**Figure K Fi**), and PLI (**Figure I Fii**), which decreased steadily across the stimulation period in all conditions (**Figure I Hi**, **Figure I Hii**)

Overall, we observed again that the effects of αCLAS on brain dynamics are dependent on the phase applied and the location of the alpha rhythm targeted.


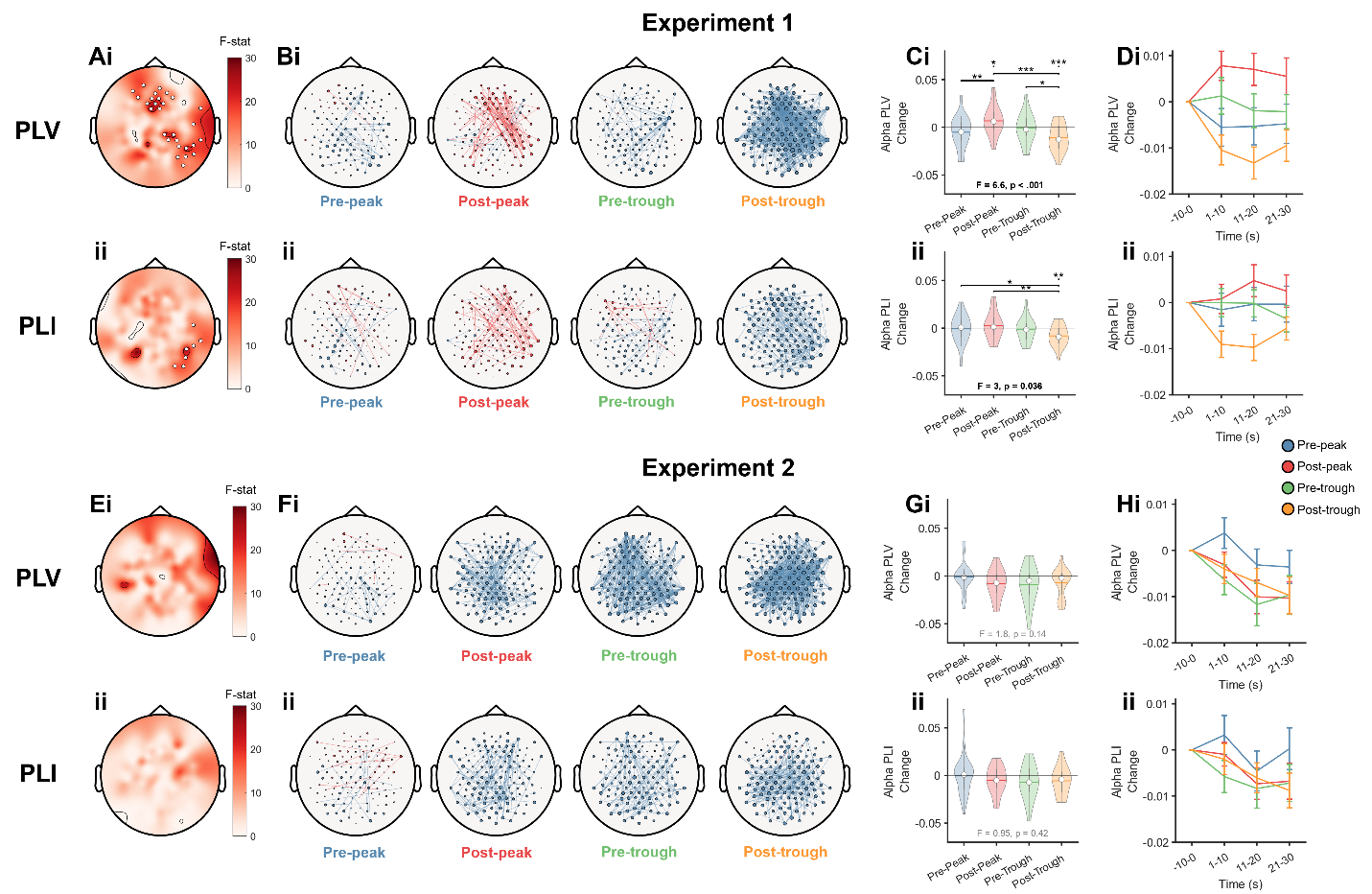


**Figure I. Stimulation-induced connectivity changes in alpha band (experiments 1 and 2) (Ai** and **Ei)** Topography of main effect of phase on average alpha band PLV for each channel as per ANOVA, for experiment 1 and 2 respectively. White marks indicate cluster-corrected p<0.05. **(Aii** and **Eii)** Topography of variance between conditions of average alpha band PLI for each channel as per ANOVA, for experiment 1 and 2 respectively. White marks indicate cluster-corrected p<0.05. **(Bi** and **Fi)** Stimulation-induced changes in alpha band PLV per condition as per t-test (compared to the ‘off’ period), lines are plotted where *p*<0.01. Red lines indicate an increase in the connectivity of that channel pair, blue lines indicate a decrease. **(Bii** and **Fii)** Stimulation-induced changes in alpha band PLI per condition as per t-test, lines are plotted where *p*<0.01. Red lines indicate an increase in the connectivity of that channel pair, blue lines indicate a decrease. **(Ci** and **Gi)** Stimulation-induced changes in alpha band PLV per condition, collapsed across the stimulation period and across all other channels and the region of interest, in experiment 1 and 2, respectively. **(Cii** and **Gii)** Stimulation-induced changes in alpha band PLI per condition, collapsed across the stimulation period and across all other channels and the region of interest, in experiment 1 and 2, respectively. For all violin plots, mixed effects models were run: [connectivity ~ condition + (1|Participant)]. Post-hoc Wald tests were run for those which showed a statistically significant effect of condition (*p* < 0.05). * *p*<0.05, ***p*<0.01, ****p*<.001. Bars between conditions indicate differences between conditions, asterisks above conditions indicate a significant difference from zero, as per one-sample t-test. **(Di** and **Hi)** Stimulation-induced changes in alpha band PLV per condition, collapsed across all other channels and the region of interest across *time*, in experiment 1 and 2, respectively. **(Dii** and **Hii)** Stimulation-induced changes in alpha band PLI per condition, collapsed across all other channels and the region of interest across *time*, in experiment 1 and 2, respectively.


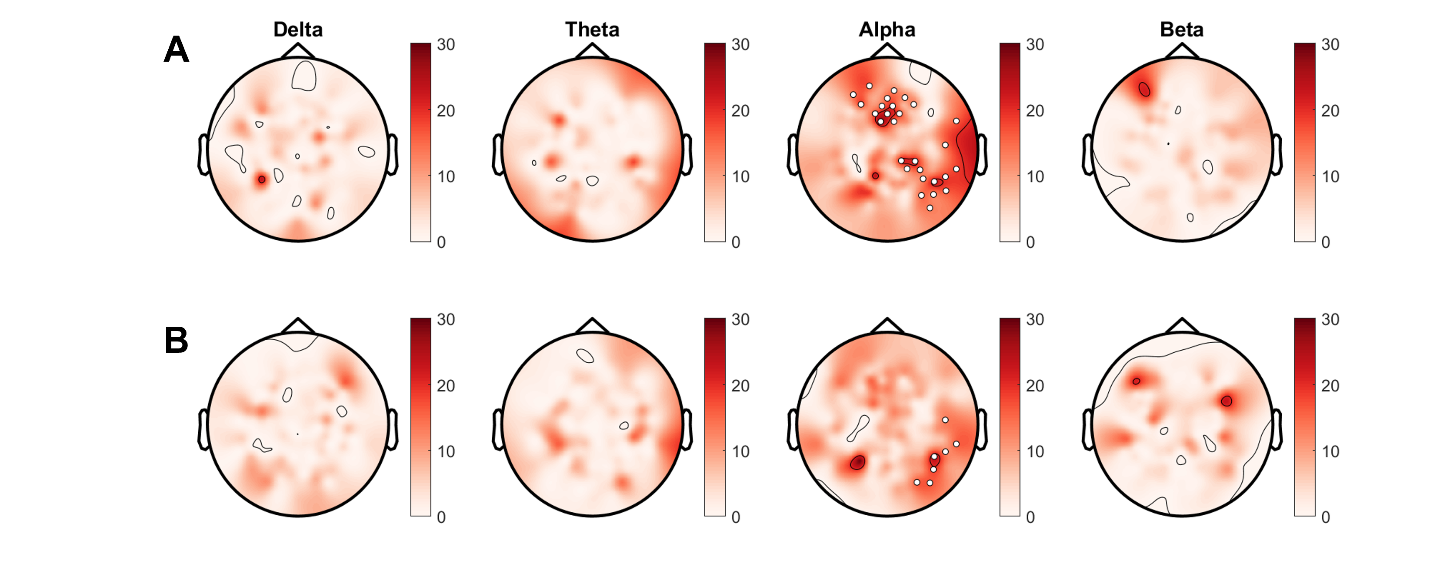


**Figure J. Topography of permutation ANOVA stats for connectivity in each frequency band in experiment 1. (A)** Phase-locking value (PLV), **(B)** phase lag index (PLI). White dots show significant main effect of phase-targeted, cluster-corrected *p* <0.05.


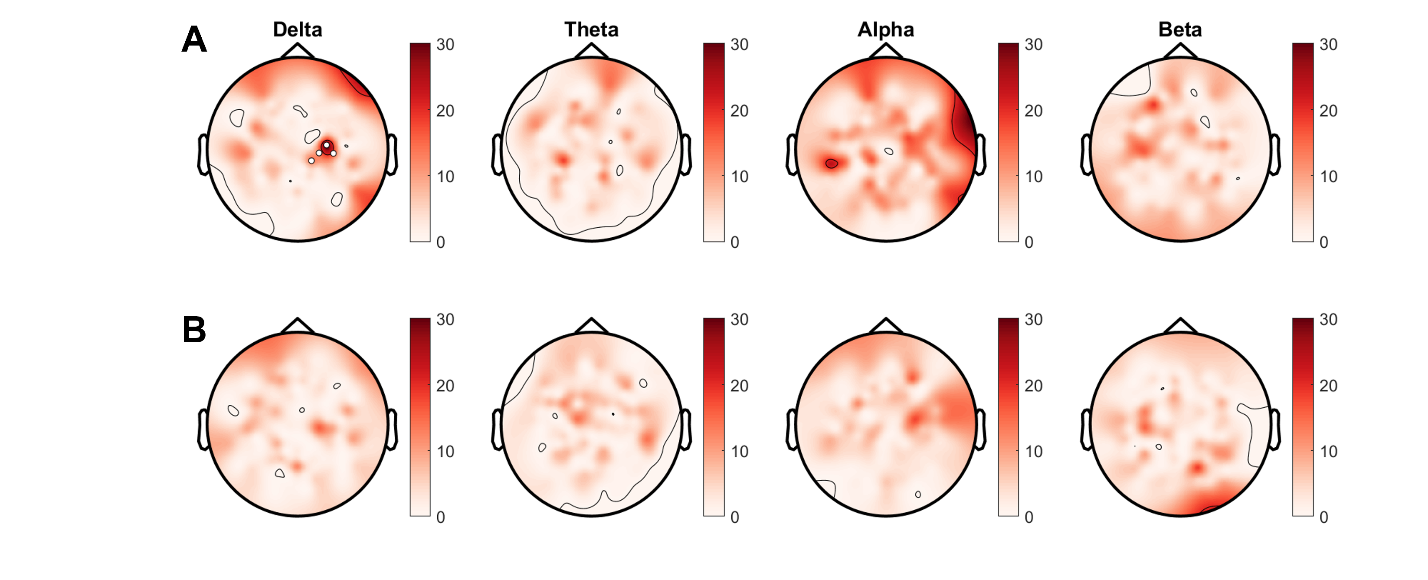


**Figure K. Topography of permutation ANOVA stats for connectivity in each frequency band in experiment 2. (A)** Phase-locking value (PLV), **(B)** phase lag index (PLI). White dots show significant main effect of phase-targeted, cluster-corrected *p* <0.05.


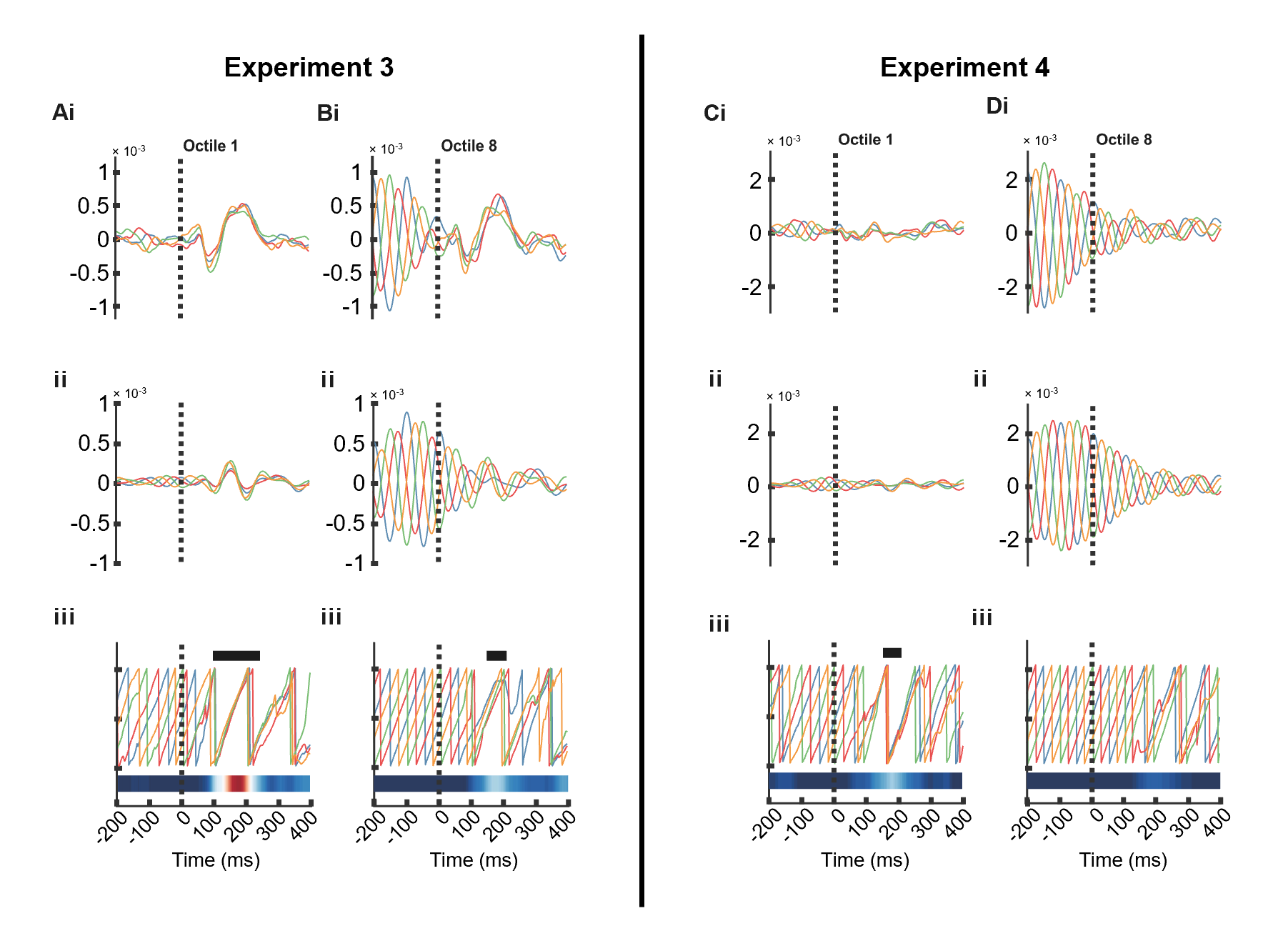


**Figure L. Auditory evoked potential (AEP) and Phase Reset**

(**A** and **C**) Lowest pre-stimulus alpha power octile at Fz in experiment 3 (**A**) and Pz in experiment 4 (**C**). (**B** and **D**) Highest pre-stimulus alpha power octile at Fz in experiment 3 (**B**) and Pz in experiment 4 (**D**). (**A, B, C, D**) **(i)** Broadband (1-40 Hz) AEP*; **ii.** Amplitude component of alpha band (7.5-12.5 Hz) endpoint-corrected Hilbert transformed AEP*; **iii.** Instantaneous phase of alpha band (7.5-12.5 Hz) from endpoint-corrected Hilbert transformed AEP**. Even at Pz, in experiment 4 there was post-stimuli phase alignment when the pre-stimulus alpha power was at its lowest amplitude (**Ciii**). * Black marks indicate ANOVA *p* <0.05. ** Black marks indicate Rayleigh test *p* <0.05, heatmap shows time series of Z-statistic. Fz and Pz electrodes from the hd-EEG system.

**
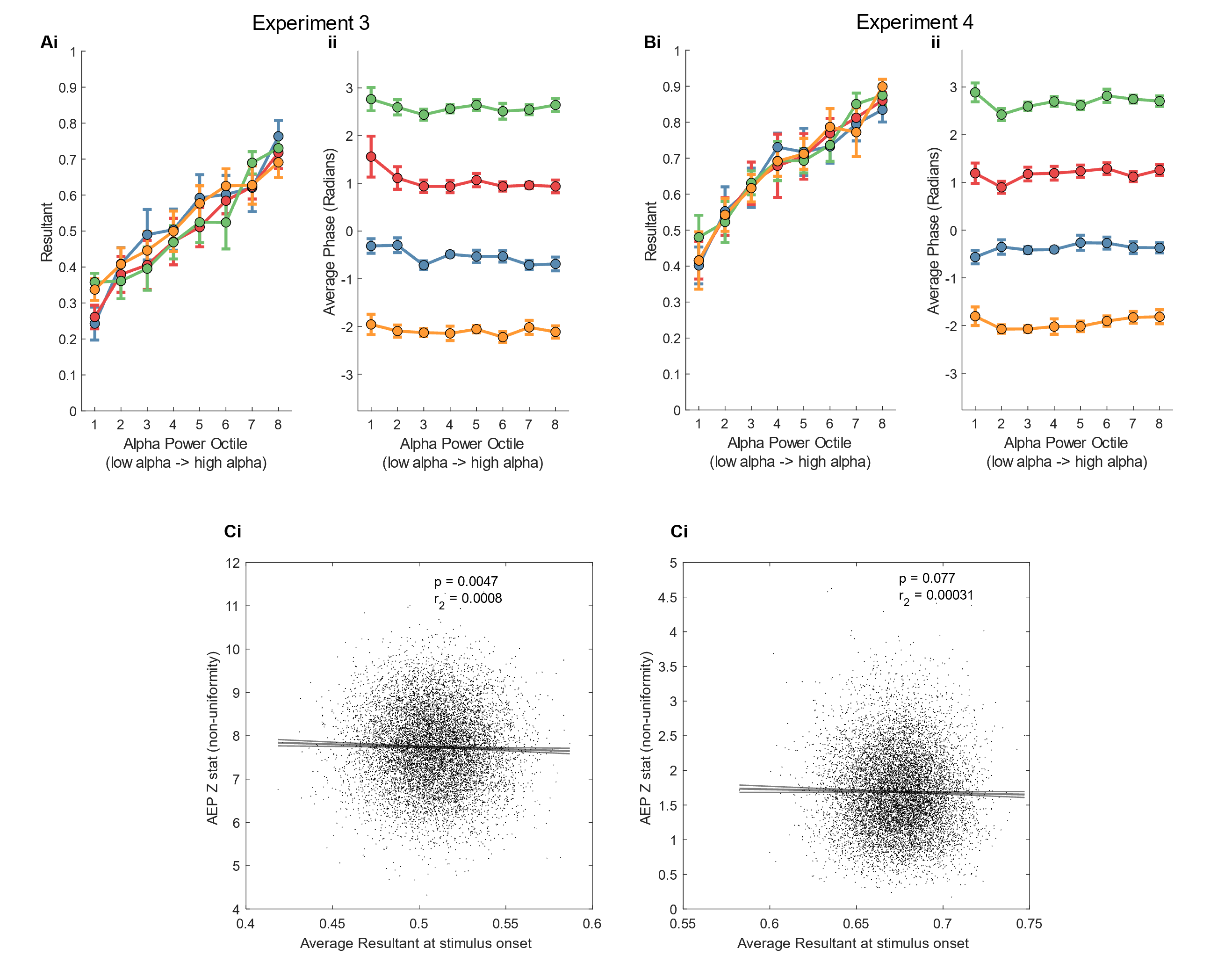
**

**Figure M. Resultant per Octile, and Resultant vs Z Stat for Experiments 3 and 4**

(**Ai** and **Bi**) There was a clear linear relationship between alpha power octile and stimulus onset resultant in each condition, in both experiments. This meant that the phase was most consistent between trials in octile 8, and least consistent between trials in octile 1. We considered that this likely resulted from the use of two independent EEG systems, since the phase-locking (ecHT) system and the hd-EEG will be in greatest agreement, regarding phase, when alpha power is high, and stimulus onset is determined only by the ecHT. We suggest that the extent of the reset should not be dependent on this onset resultant. (**Aii** and **Bii**) The average phase angle was highly consistent across octiles for both experiments.

(**C**) Here we tested the extent by which the phase reset was related to the resultant at stimulus onset. We took 10,000 samples of 20 trials from each condition, computing stimulus onset resultant, averaging across conditions, and plotting this against auditory-evoked Z statistic. We found a statistically significant (in experiment 1, but not experiment 2), but very weak relationship (R^2^ values <0.001), and hence confirmed our intuition that z-stat is not strongly dependent on onset resultant.

**Table C | Number of trials per phase bin (experiments 3 and 4).** Phase estimates were used to sort all evoked potentials by stimulus onset phase, into ten 36° bins. N indicates number of participants.

| **Trials in each phase bin** | | | | |  | | | | | | |  | |
| --- | --- | --- | --- | --- | --- | --- | --- | --- | --- | --- | --- | --- | --- |
| **Experiment/Location** | | **Phase (°)** | | | | | | | | | | | |
|  |  | **0** | **36** | **72** | | **108** | **144** | **180** | **216** | **252** | **288** | | **324** |
| **3/Fz (N=8)** | **Mean** | 66.8 | 80 | 74.8 | | 71.5 | 71.8 | 67.1 | 80 | 76.3 | 77.8 | | 74.1 |
|  | **SD** | 8.0 | 6.7 | 6.7 | | 8.0 | 9.4 | 10.6 | 10.1 | 9.6 | 6.8 | | 8.0 |
| **4/Pz (N=7)** | **Mean** | 76.9 | 67.1 | 80.4 | | 67.4 | 74.3 | 70.1 | 71.7 | 69 | 68.1 | | 76.9 |
|  | **SD** | 14.1 | 17.4 | 8.4 | | 11.8 | 12.7 | 17.8 | 14.2 | 12.0 | 14.0 | | 14.1 |


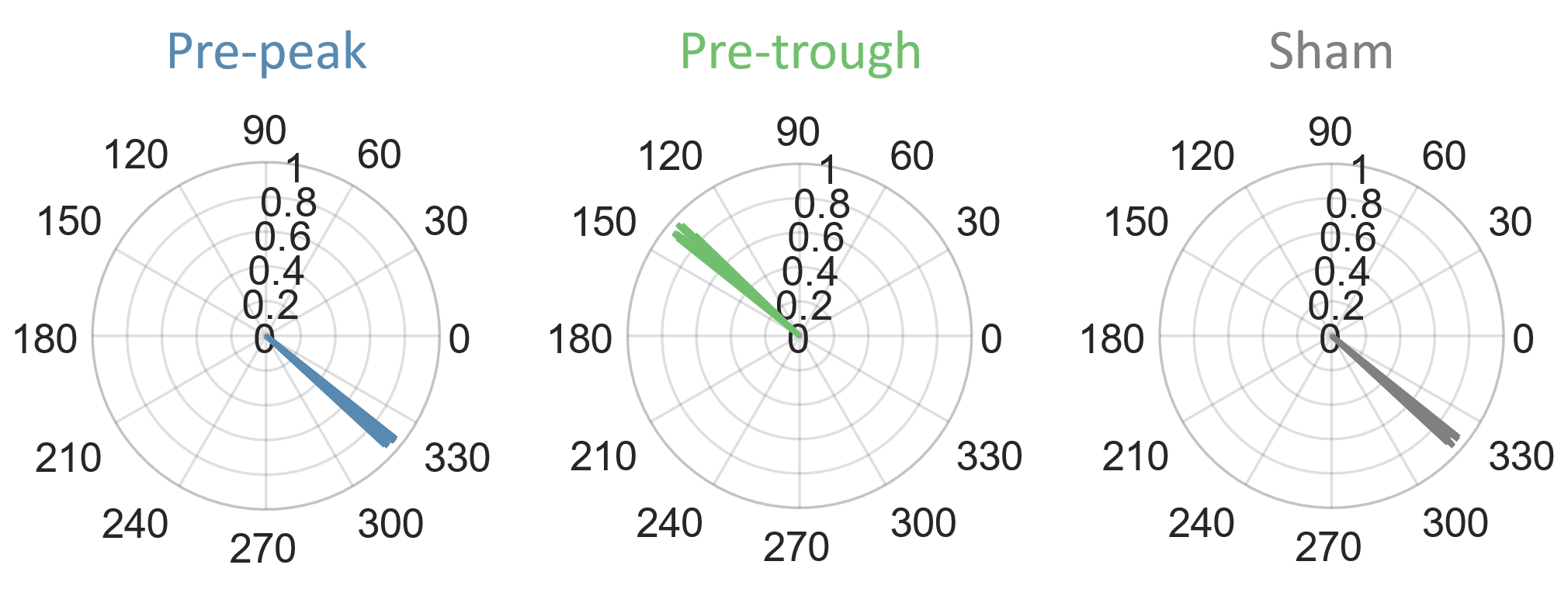


**Figure N. Phase accuracy plots for the ecHT electrode (in Fz) in the three conditions in Experiment 5.** Each line represents a participant, length of line indicates resultant (between 0 and 1). Phase accuracy is high in all three conditions. Phases are the same for pre-peak and sham. During sham, markers were recorded for each sound stimulus, but the volume was zero.


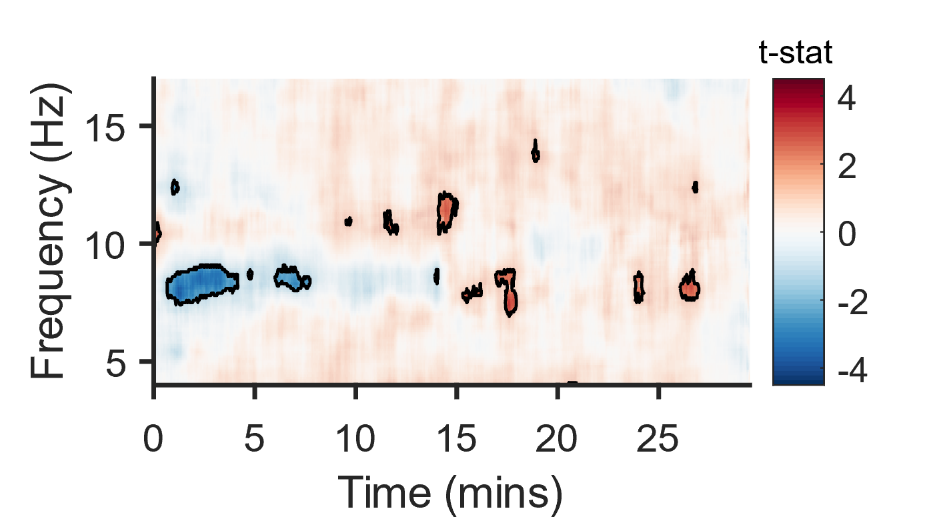


**Figure O. Effects of stimulation phase on power and frequency at Fz for the whole duration of the nap opportunity in experiment 5.** Time-frequency representation of differences between stimulation conditions across the 30-minute nap opportunity, as per paired t-tests. Red colours indicate pre-peak>pre-trough and blue indicates pre-preak<pre-trough. Black lines outline statistically significant clusters.


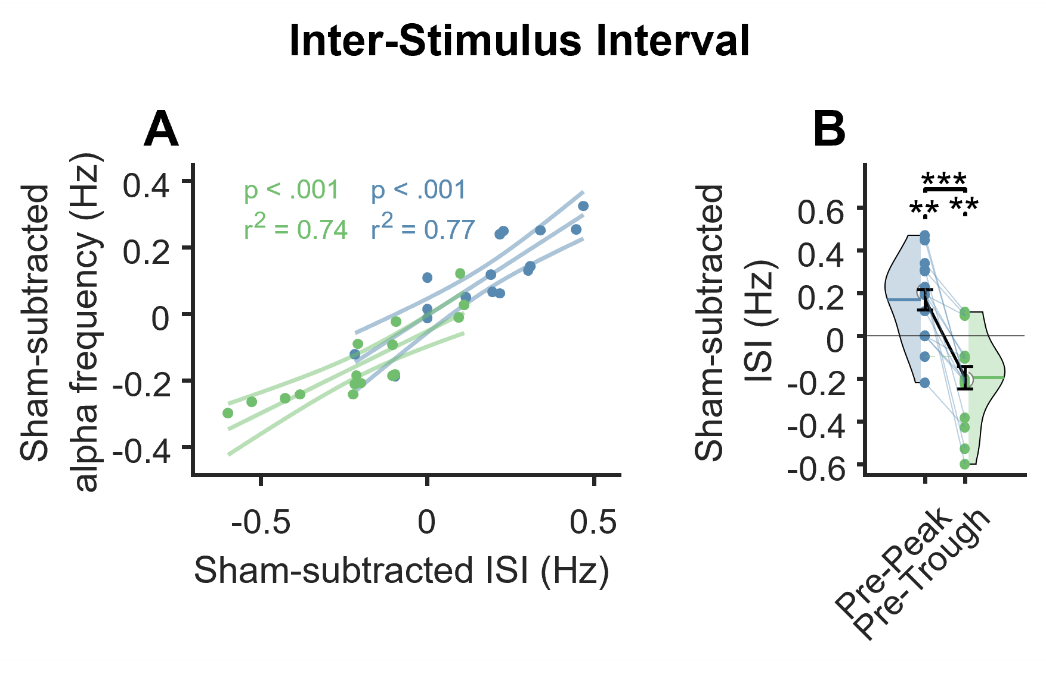


**Figure P. Inter-stimulus interval (ISI) and relation to individual alpha frequency for experiment 5. (A)** Sham-subtracted ISI vs sham-subtracted alpha frequency. Lines and statistics derived from simple linear regression for each condition. The ISI between pulses of sounds showed a strong linear relationship with alpha frequency as observed in experiment 1 (**Figure 1D**). In a truly closed-loop experiment, it can become difficult to distinguish the direction of causality – did shorter ISI’s speed up the brain’s rhythms or did faster brain rhythms lead to shorter ISI’s? Using the data from the sham condition, in which sound triggers were locked to pre-peak, but no sound was played, allowed us to disentangle this conundrum. These dummy sound pulses were locked to the same phase as the pre-peak condition (**Figure N**), but the ISIs differed, indicating that the ISI was indeed dependent on the brain’s responses to sound, as opposed to the particular phase targeted. Furthermore, the distribution of ISIs differed between vigilance states further suggesting that the ISI and therefore the closed-loop algorithm used is dependent on the brain’s physiology (see **Figure Q**). **(B)** average sham-subtracted ISI for pre-peak and pre-trough conditions. *** *p* < 0.001, ** *p* < 0.01, * *p* < 0.05, t-tests. Blue represents pre-peak and green represents pre-trough.


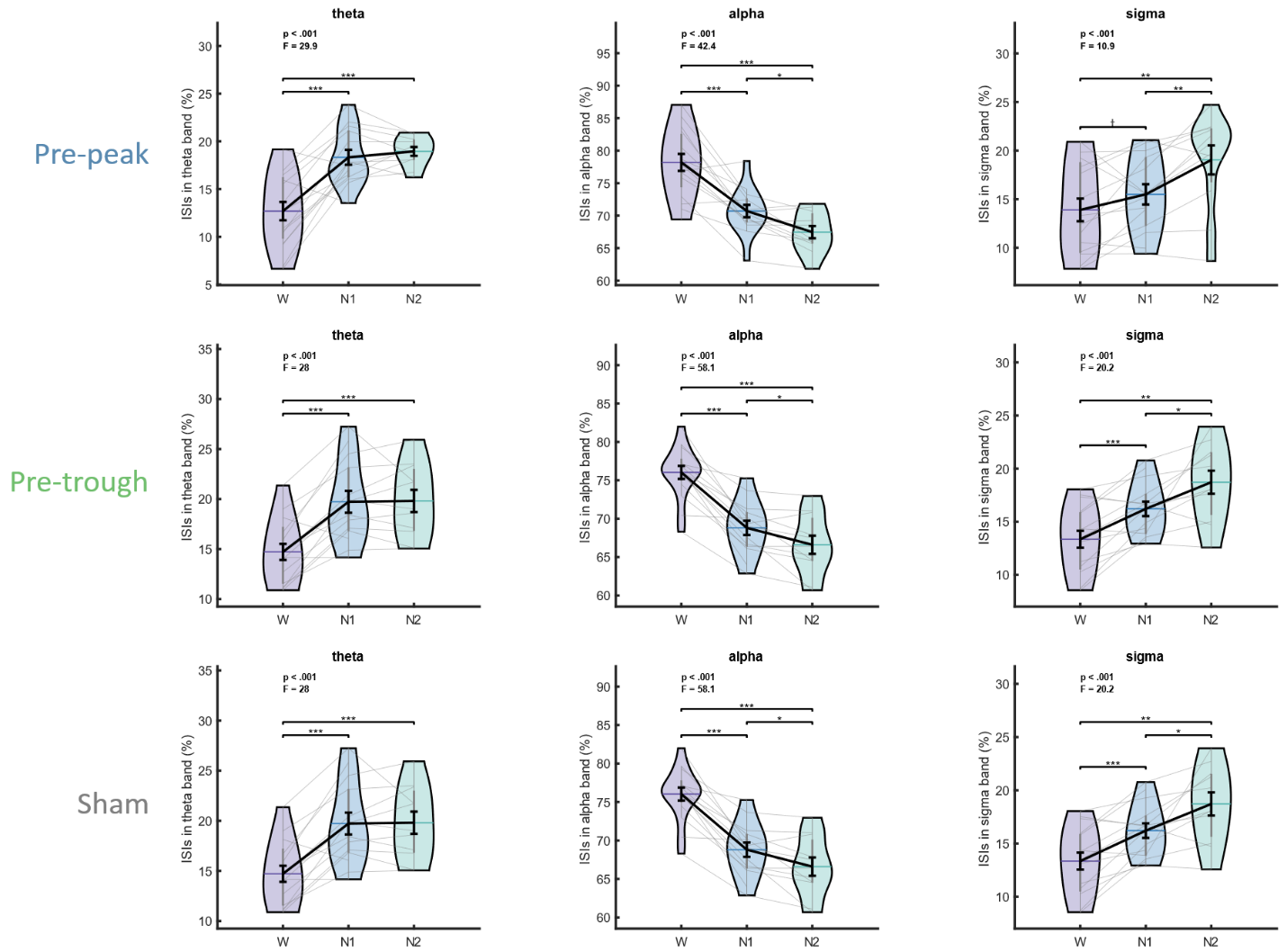


**Figure Q. Inter stimulus intervals per sleep stage.** ISIs of the sounds delivered in the pre-peak and pre-trough conditions and dummy sounds in the sham condition (i.e., no sound output) were investigated for each vigilance state (awake – W, N1 and N2 sleep). As participants transitioned from awake to N1 and N2 during experiment 5, the main frequencies detected in the phase-locking electrode also changed (alpha is more predominant during wakefulness, theta during N1 and sigma during N2). The shift in ISIs detected per vigilance stage for each stimulation condition indicates that the αCLAS algorithm followed brain activity. Violin plots show the percentage of ISI’s in each frequency band, in each sleep stage, in each condition. Stats indicate output of linear mixed effects model [ISI_percentage ~ sleep_stage + (1|participant)]. * p <0.05, ** p<0.01, ***p<0.001.


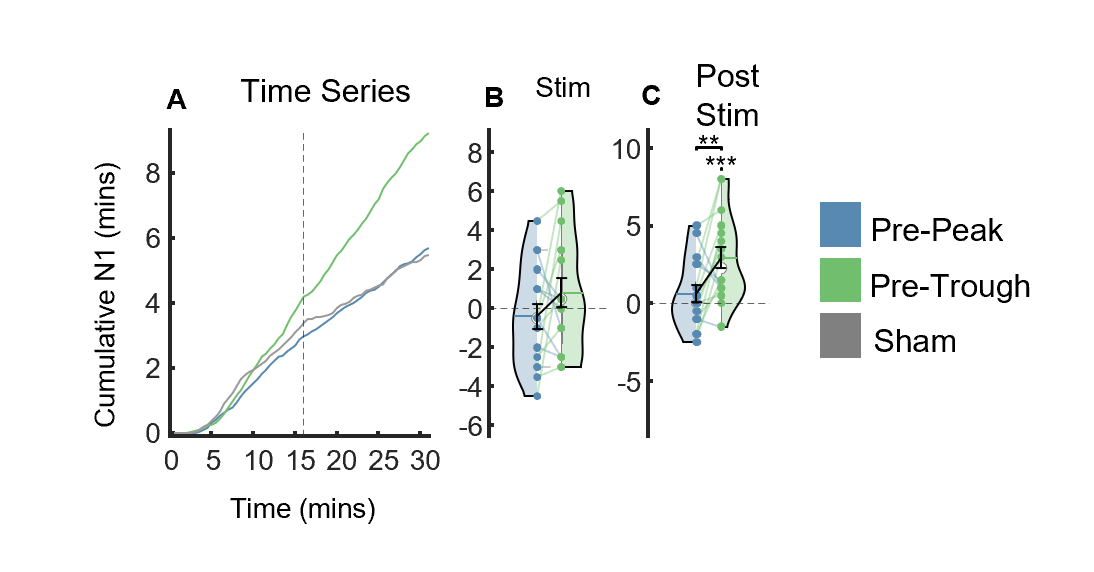


**Figure R. Cumulative N1 during experiment 5.** **(A)** Cumulative N1 sleep timeseries. Dashed line indicates end of stimulation. The plot shows that cumulative N1 sleep trailed off in pre-peak and sham conditions, as participants transitioned to deeper stages of sleep, whilst in pre-trough the amount of N1 continued to accumulate, with significantly more N1 sleep seen post-stimulation (see panel C). **(B)** Sham-subtracted time in N1 sleep collapsed across stimulation period. **(C)** Sham-subtracted time in N1 sleep collapsed across post-stimulation period. Note: statistics were run prior to sham subtraction. Post-hoc comparisons were only carried out when a main effect of condition was seen in LME. Significance bars between violins indicate a difference between stimulation conditions, significance marks over violins indicate a difference from sham. *** *p* < 0.001, ** *p* < 0.01, * *p* < 0.05, † *p* < 0.1, t-tests. Each violin shows a dot per participant, per condition, horizontal lines indicate the mean.


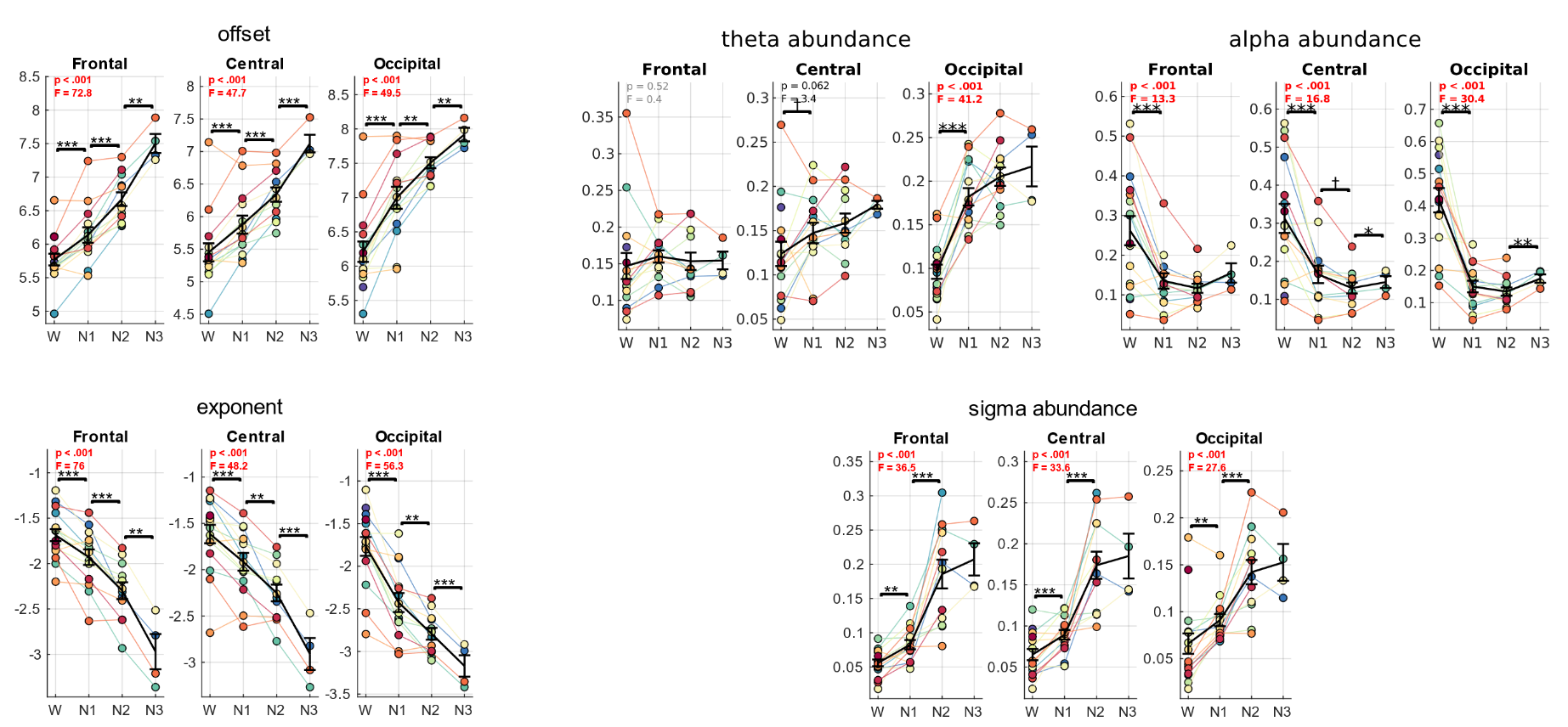

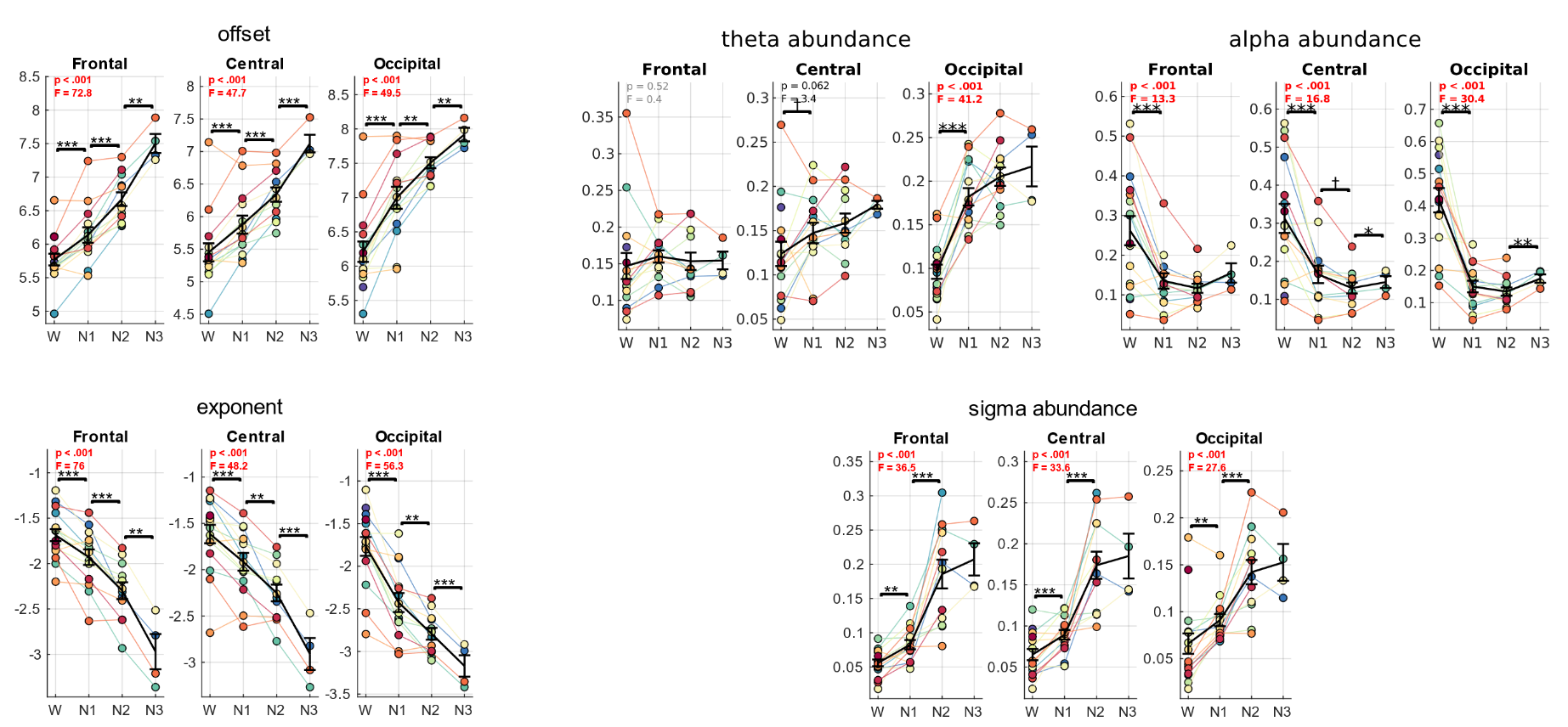


**Figure S – eBOSC features per sleep stage for the sham condition.** Averages are shown for each feature, each region, each participant (coloured dots), for the sham condition. Stats indicate output of linear mixed effects model [eBOSC_feature ~ sleep_stage + (1|participant)]. * p <0.05, ** p<0.01, ***p<0.001.

**Figure T. Time series for each eBOSC feature in experiment 5.** Average time series are shown for each feature, each region, for each condition. Data was smoothed using a 2-minute moving mean window. Dashed line indicates end of stimulation. Timecourses are represented in blue for pre-peak condition, green for pre-trough condition and grey for sham condition. ‘Frontal’ refers to the averaged data from channels F3 and F4, ‘Central’ from channels C3 and C4 and ‘Occipital’ from channels O1 and O2, all from the PSG system.


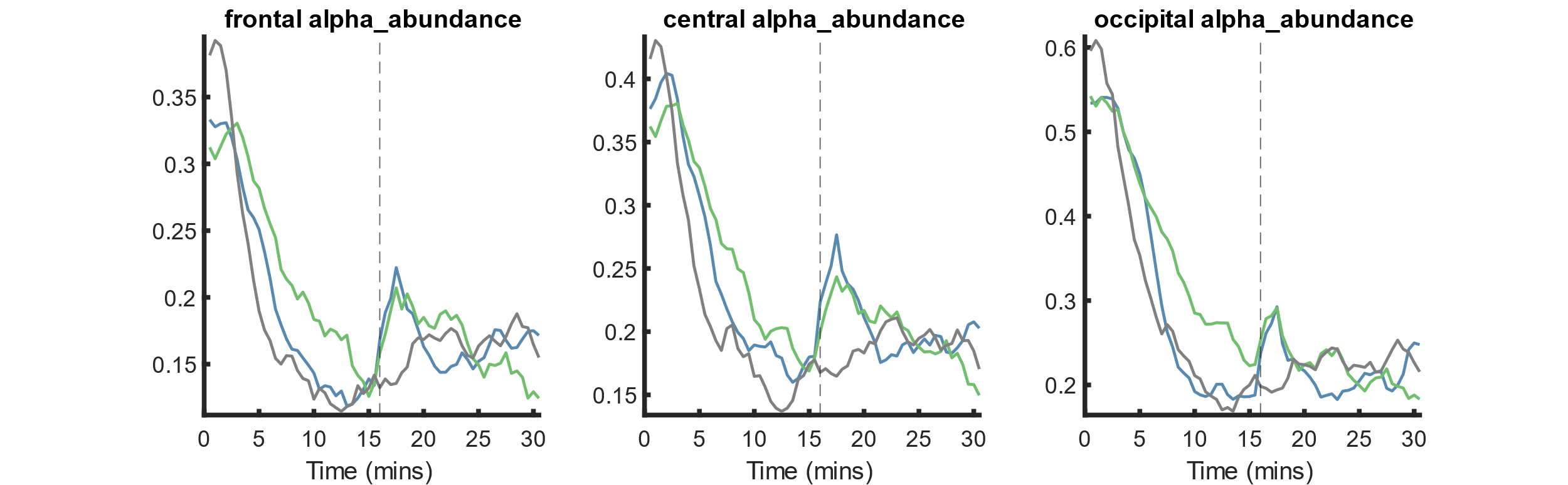

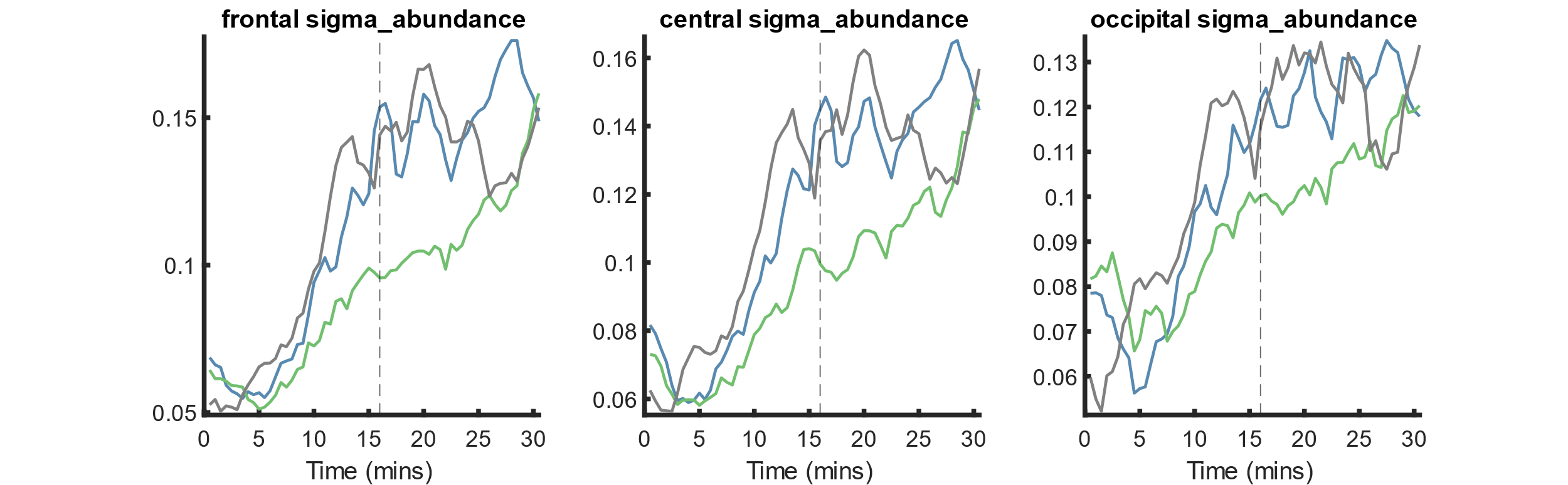

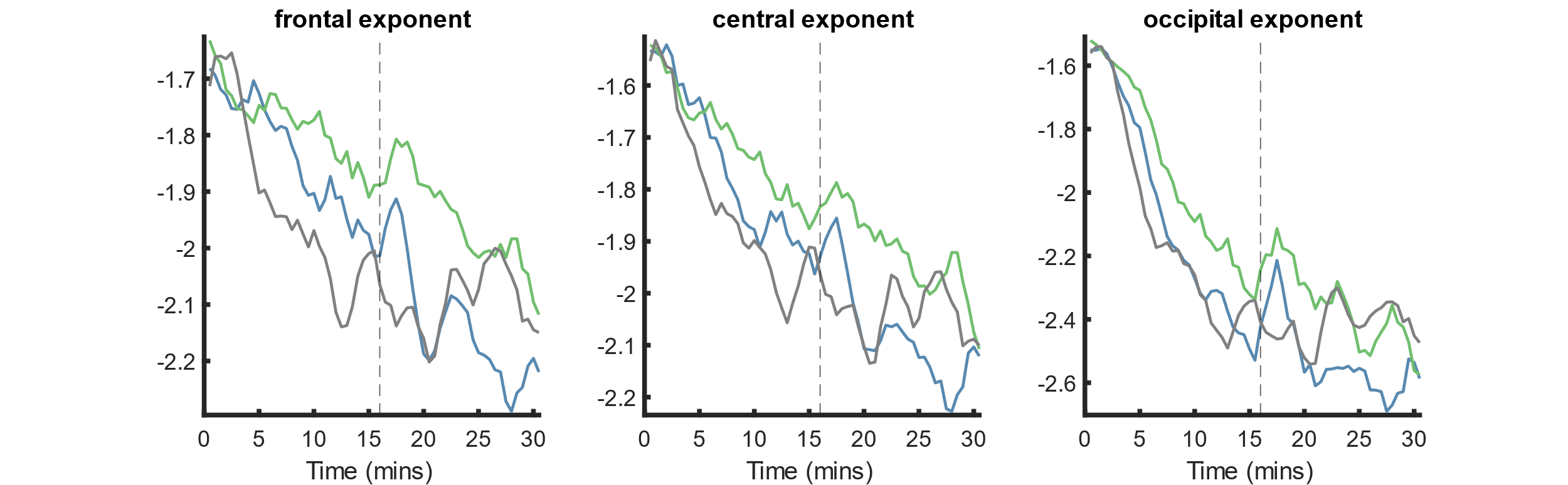

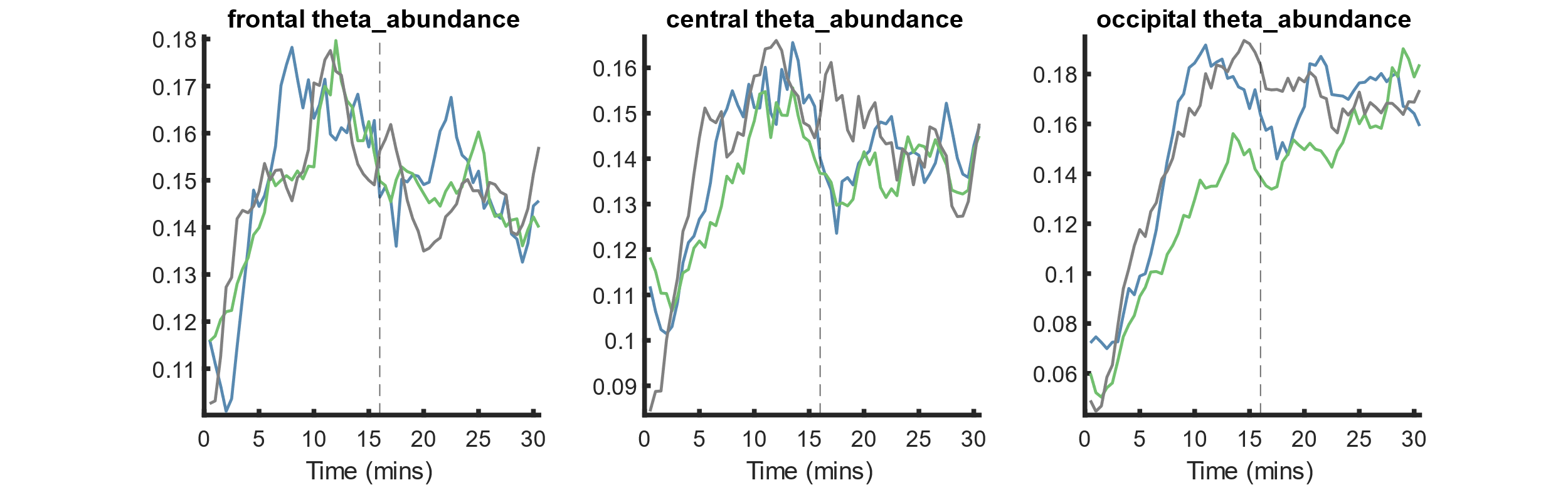

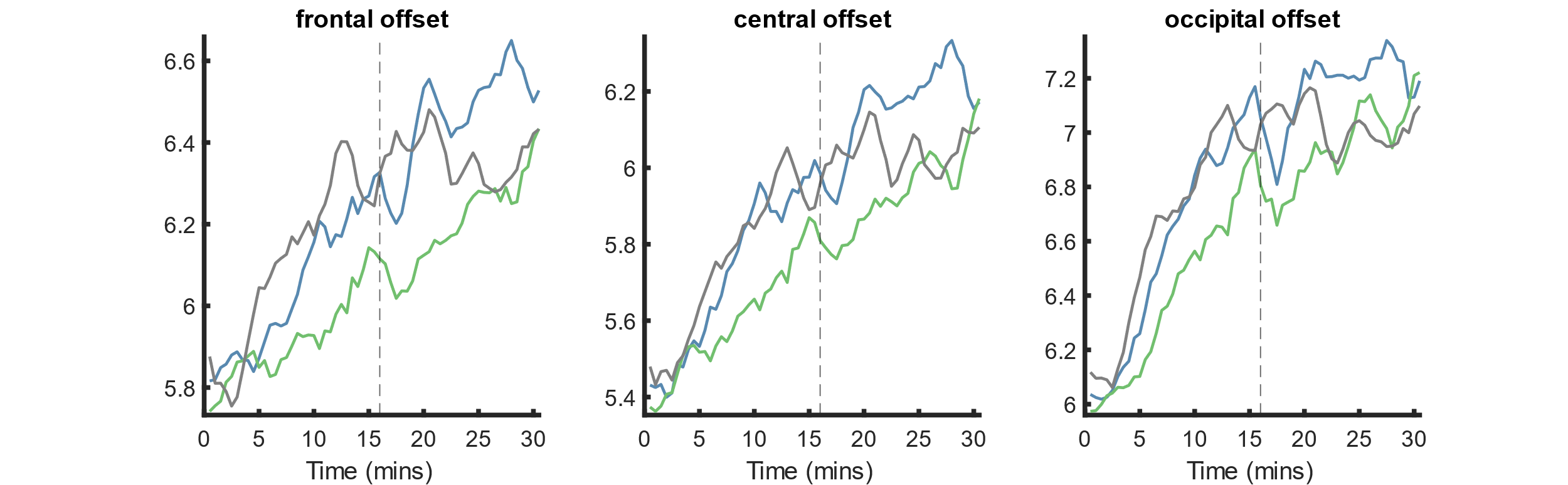


**Pre-peak**

**Pre-trough**

**Sham**


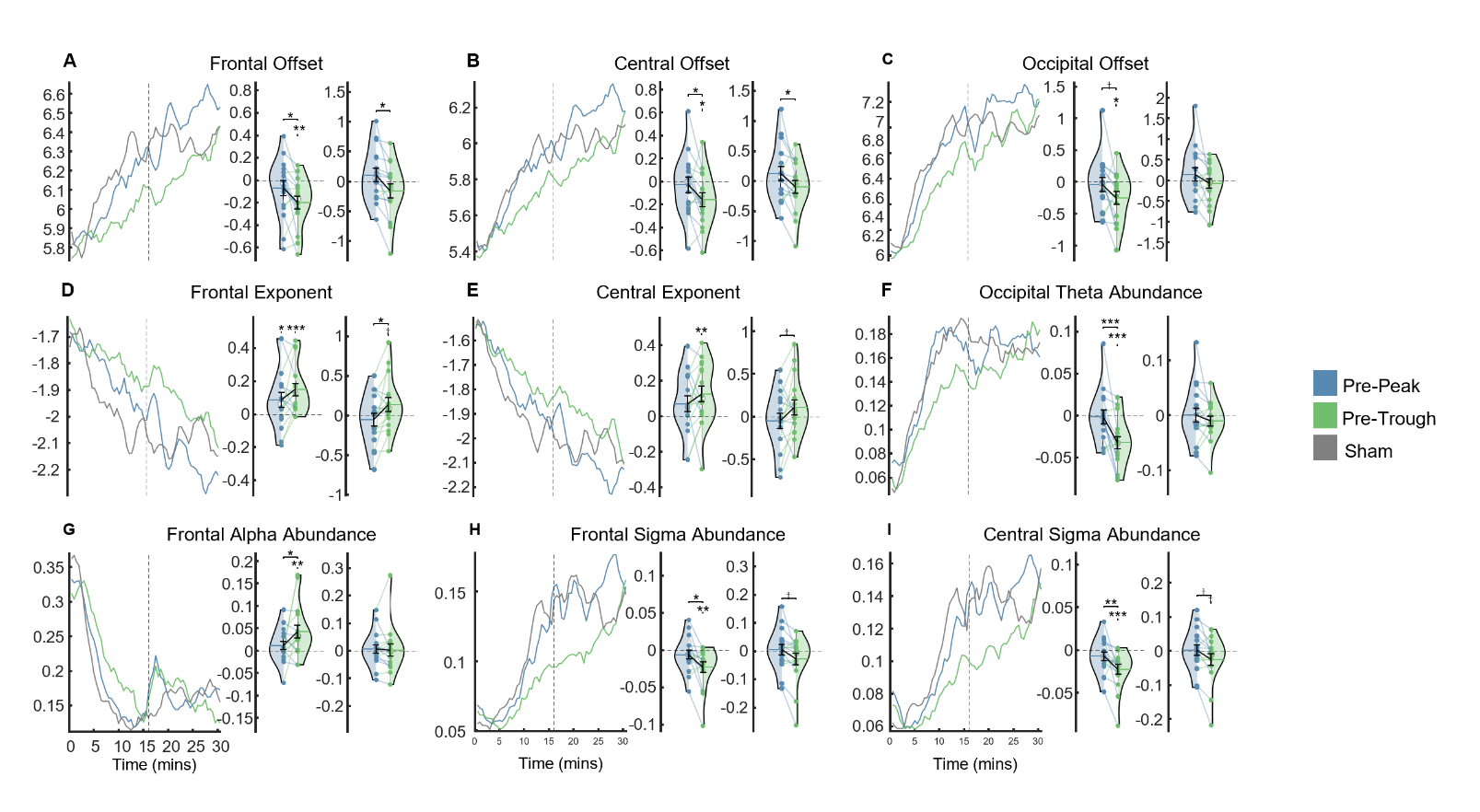


**Figure U. All eBOSC features showing a main effect of condition. (A-I)** Timeseries of EEG eBOSC features (left) averaged across participants. Sham-subtracted features collapsed across stimulation period (middle) and post-stimulation period (right). ‘Frontal’ refers to the averaged data from channels F3 and F4, ‘Central’ from channels C3 and C4 and ‘Occipital’ from channels O1 and O2, all from the PSG system. Note: statistics were run prior to sham subtraction. Post-hoc comparisons were only carried out when a main effect of condition was seen in LME. Significance bars between violins indicate a difference between stimulation conditions, significance marks over violins indicate a difference from sham. *** *p* < 0.001, ** *p* < 0.01, * *p* < 0.05, † *p* < 0.1, t-tests. Each violin shows a dot per participant, per condition, horizontal lines indicate the mean.

**Table D | Sleep scoring**

Duration in minutes of each vigilance state (wake, sleep N1, N2 and N3) for each participant, in each condition. Values are broken down into stimulation, post-stimulation, and whole nap periods.

| **Participant** | **Condition** | **Stim Wake** | **Stim N1** | **Stim N2** | **Stim N3** | **Post-Stim Wake** | **Post-Stim N1** | **Post-Stim N2** | **Post-Stim N3** | **Whole Nap Wake** | **Whole Nap N1** | **Whole Nap N2** | **Whole Nap N3** |
| --- | --- | --- | --- | --- | --- | --- | --- | --- | --- | --- | --- | --- | --- |
| **1** | Pre-Peak | 4.5 | 3 | 7.5 | 0 | 1 | 2.5 | 10.5 | 1 | 6.5 | 5.5 | 18 | 1 |
|  | Post-Peak | 12 | 3 | 0 | 0 | 6 | 8 | 1 | 0 | 19 | 11 | 1 | 0 |
|  | Sham | 15 | 0 | 0 | 0 | 15 | 0 | 0 | 0 | 31 | 0 | 0 | 0 |
| **2** | Pre-Peak | 3.5 | 3.5 | 8 | 0 | 0.5 | 1.5 | 9 | 4 | 5 | 5 | 17 | 4 |
|  | Post-Peak | 5.5 | 3 | 6.5 | 0 | 1 | 5 | 9 | 0 | 7.5 | 8 | 15.5 | 0 |
|  | Sham | 3.5 | 2.5 | 9 | 0 | 6 | 3.5 | 5 | 0.5 | 10.5 | 6 | 14 | 0.5 |
| **3** | Pre-Peak | 6.5 | 2 | 6.5 | 0 | 3.5 | 1.5 | 10 | 0 | 11 | 3.5 | 16.5 | 0 |
|  | Post-Peak | 13.5 | 1.5 | 0 | 0 | 6 | 8.5 | 0.5 | 0 | 20.5 | 10 | 0.5 | 0 |
|  | Sham | 5 | 4 | 6 | 0 | 0 | 0.5 | 14.5 | 0 | 6 | 4.5 | 20.5 | 0 |
| **4** | Pre-Peak | 2 | 1 | 4 | 8 | 0 | 0 | 0.5 | 14.5 | 3 | 1 | 4.5 | 22.5 |
|  | Post-Peak | 4.5 | 8.5 | 2 | 0 | 0 | 0.5 | 14.5 | 0 | 5.5 | 9 | 16.5 | 0 |
|  | Sham | 3 | 4 | 8 | 0 | 0 | 0 | 8 | 7 | 4 | 4 | 16 | 7 |
| **5** | Pre-Peak | 4 | 4 | 7 | 0 | 3 | 4 | 8 | 0 | 8 | 8 | 15 | 0 |
|  | Post-Peak | 12 | 2 | 1 | 0 | 3 | 6 | 6 | 0 | 16 | 8 | 7 | 0 |
|  | Sham | 4.5 | 4.5 | 6 | 0 | 1 | 5 | 9 | 0 | 6.5 | 9.5 | 15 | 0 |
| **6** | Pre-Peak | 15 | 0 | 0 | 0 | 6.5 | 8.5 | 0 | 0 | 22.5 | 8.5 | 0 | 0 |
|  | Post-Peak | 8 | 7 | 0 | 0 | 1.5 | 9.5 | 4 | 0 | 10.5 | 16.5 | 4 | 0 |
|  | Sham | 8.5 | 4.5 | 2 | 0 | 1 | 3.5 | 10.5 | 0 | 10.5 | 8 | 12.5 | 0 |
| **7** | Pre-Peak | 13 | 2 | 0 | 0 | 4.5 | 1.5 | 9 | 0 | 18.5 | 3.5 | 9 | 0 |
|  | Post-Peak | 13 | 2 | 0 | 0 | 2.5 | 5 | 7.5 | 0 | 16.5 | 7 | 7.5 | 0 |
|  | Sham | 10 | 5 | 0 | 0 | 0 | 1.5 | 13.5 | 0 | 11 | 6.5 | 13.5 | 0 |
| **8** | Pre-Peak | 11.5 | 3.5 | 0 | 0 | 5 | 6.5 | 3.5 | 0 | 17.5 | 10 | 3.5 | 0 |
|  | Post-Peak | 13 | 2 | 0 | 0 | 5.5 | 4.5 | 5 | 0 | 19.5 | 6.5 | 5 | 0 |
|  | Sham | 13.5 | 1.5 | 0 | 0 | 11 | 3.5 | 0.5 | 0 | 25.5 | 5 | 0.5 | 0 |
| **9** | Pre-Peak | 6 | 5.5 | 3.5 | 0 | 0 | 0.5 | 7 | 7.5 | 7 | 6 | 10.5 | 7.5 |
|  | Post-Peak | 12.5 | 1.5 | 1 | 0 | 1.5 | 7 | 6.5 | 0 | 15 | 8.5 | 7.5 | 0 |
|  | Sham | 6 | 2.5 | 6.5 | 0 | 0.5 | 2.5 | 9.5 | 2.5 | 7.5 | 5 | 16 | 2.5 |
| **10** | Pre-Peak | 7.5 | 4 | 3.5 | 0 | 8.5 | 0 | 6.5 | 0 | 17 | 4 | 10 | 0 |
|  | Post-Peak | 2.5 | 5.5 | 7 | 0 | 3 | 5.5 | 6.5 | 0 | 6.5 | 11 | 13.5 | 0 |
|  | Sham | 4 | 6.5 | 4.5 | 0 | 3.5 | 2.5 | 9 | 0 | 8.5 | 9 | 13.5 | 0 |
| **11** | Pre-Peak | 15 | 0 | 0 | 0 | 9.5 | 5 | 0.5 | 0 | 25.5 | 5 | 0.5 | 0 |
|  | Post-Peak | 11.5 | 3.5 | 0 | 0 | 13.5 | 1.5 | 0 | 0 | 26 | 5 | 0 | 0 |
|  | Sham | 14.5 | 0.5 | 0 | 0 | 14.5 | 0.5 | 0 | 0 | 30 | 1 | 0 | 0 |
| **12** | Pre-Peak | 4.5 | 2.5 | 8 | 0 | 0.5 | 3.5 | 11 | 0 | 6 | 6 | 19 | 0 |
|  | Post-Peak | 6 | 3 | 6 | 0 | 4.5 | 3 | 7.5 | 0 | 11.5 | 6 | 13.5 | 0 |
|  | Sham | 4.5 | 6 | 4.5 | 0 | 6 | 4.5 | 4.5 | 0 | 11.5 | 10.5 | 9 | 0 |
| **13** | Pre-Peak | 3.5 | 2.5 | 9 | 0 | 0 | 0 | 4 | 11 | 4.5 | 2.5 | 13 | 11 |
|  | Post-Peak | 5.5 | 4 | 5.5 | 0 | 0 | 0 | 2.5 | 12.5 | 6.5 | 4 | 8 | 12.5 |
|  | Sham | 2 | 3.5 | 9 | 0.5 | 0 | 0 | 1.5 | 13.5 | 3 | 3.5 | 10.5 | 14 |
| **14** | Pre-Peak | 4.5 | 4 | 6.5 | 0 | 4.5 | 4.5 | 6 | 0 | 10 | 8.5 | 12.5 | 0 |
|  | Post-Peak | 4.5 | 10.5 | 0 | 0 | 9 | 3.5 | 2.5 | 0 | 14.5 | 14 | 2.5 | 0 |
|  | Sham | 5 | 4.5 | 5.5 | 0 | 0 | 2 | 13 | 0 | 6 | 6.5 | 18.5 | 0 |
| **15** | Pre-Peak | 5.5 | 9 | 0.5 | 0 | 3.5 | 3.5 | 4.5 | 3.5 | 10 | 12.5 | 5 | 3.5 |
|  | Post-Peak | 4.5 | 10 | 0.5 | 0 | 4 | 8 | 3 | 0 | 9.5 | 18 | 3.5 | 0 |
|  | Sham | 6.5 | 4.5 | 4 | 0 | 8.5 | 4 | 2.5 | 0 | 16 | 8.5 | 6.5 | 0 |
| **16** | Pre-Peak | 14 | 1 | 0 | 0 | 14.5 | 0.5 | 0 | 0 | 29.5 | 1.5 | 0 | 0 |
|  | Post-Peak | 15 | 0 | 0 | 0 | 10 | 5 | 0 | 0 | 26 | 5 | 0 | 0 |
|  | Sham | 15 | 0 | 0 | 0 | 15 | 0 | 0 | 0 | 31 | 0 | 0 | 0 |

**Tables E and F | eBOSC statistics**

Tables show results of linear mixed-effects models of the form: [eBOSC_feature ~ condition + (1|participant)]. Where a main effect of condition was found (*p* <0.05), post-hoc contrasts were carried out between estimated means of model. Df, degrees of freedom; Df.res, Residual Degrees of Freedom; F, F-test statistic; p, p-value; x, not computed.

| **Table E eBOSC statistics from stimulation period** | | | | | |
| --- | --- | --- | --- | --- | --- |
|  | Df | Df.res | F | p |  |
| 'frontal_offset' | 2 | 45 | 5.923954 | 0.005202 |  |
| Post-hoc contrasts | B | SE | df | t | p |
| pre_peak - pre_trough | 0.127973 | 6.063338 | 45 | 2.206821 | 0.03247 |
| pre_peak - sham | -0.06867 | 6.063338 | 45 | 1.184249 | 0.24253 |
| pre_trough - sham | -0.19665 | 6.063338 | 45 | 3.39107 | 0.00146 |
|  | Df | Df.res | F | p |  |
| 'frontal_exponent' | 2 | 45 | 6.924841 | 0.002388 |  |
| Post-hoc contrasts | B | SE | df | t | p |
| pre_peak - pre_trough | -0.06163 | -1.85594 | 45 | 1.530077 | 0.133 |
| pre_peak - sham | 0.087522 | -1.85594 | 45 | 2.172888 | 0.03509 |
| pre_trough - sham | 0.149152 | -1.85594 | 45 | 3.702964 | 0.00058 |
|  | Df | Df.res | F | p |  |
| 'frontal_theta_abundance' | 2 | 45 | 0.156121 | 0.855917 |  |
| Post-hoc contrasts | B | SE | df | t | p |
| pre_peak - pre_trough | X | X | X | X | X |
| pre_peak - sham | X | X | X | X | X |
| pre_trough - sham | X | X | X | X | X |
|  | Df | Df.res | F | p |  |
| 'frontal_alpha_abundance' | 2 | 45 | 5.471691 | 0.007463 |  |
| Post-hoc contrasts | B | SE | df | t | p |
| pre_peak - pre_trough | -0.03095 | 0.184084 | 45 | 2.338787 | 0.02385 |
| pre_peak - sham | 0.011338 | 0.184084 | 45 | 0.856709 | 0.39615 |
| pre_trough - sham | 0.042291 | 0.184084 | 45 | 3.195496 | 0.00255 |
|  | Df | Df.res | F | p |  |
| 'frontal_sigma_abundance' | 2 | 45 | 6.631643 | 0.002992 |  |
| Post-hoc contrasts | B | SE | df | t | p |
| pre_peak - pre_trough | 0.016751 | 0.088352 | 45 | 2.58685 | 0.01299 |
| pre_peak - sham | -0.006 | 0.088352 | 45 | 0.926625 | 0.35906 |
| pre_trough - sham | -0.02275 | 0.088352 | 45 | 3.513475 | 0.00102 |
|  | Df | Df.res | F | p |  |
| 'central_offset' | 2 | 45 | 3.873805 | 0.028036 |  |
| Post-hoc contrasts | B | SE | df | t | p |
| pre_peak - pre_trough | 0.131816 | 5.75134 | 45 | 2.146797 | 0.03724 |
| pre_peak - sham | -0.0283 | 5.75134 | 45 | 0.460928 | 0.64707 |
| pre_trough - sham | -0.16012 | 5.75134 | 45 | 2.607724 | 0.01232 |
|  | Df | Df.res | F | p |  |
| 'central_exponent' | 2 | 45 | 4.061698 | 0.023897 |  |
| Post-hoc contrasts | B | SE | df | t | p |
| pre_peak - pre_trough | -0.05631 | -1.78099 | 45 | 1.270906 | 0.21029 |
| pre_peak - sham | 0.069729 | -1.78099 | 45 | 1.573878 | 0.12252 |
| pre_trough - sham | 0.126035 | -1.78099 | 45 | 2.844784 | 0.00667 |
|  | Df | Df.res | F | p |  |
| 'central_theta_abundance' | 2 | 45 | 1.547957 | 0.223794 |  |
| Post-hoc contrasts | B | SE | df | t | p |
| pre_peak - pre_trough | X | X | X | X | X |
| pre_peak - sham | X | X | X | X | X |
| pre_trough - sham | X | X | X | X | X |
|  | Df | Df.res | F | p |  |
| 'central_alpha_abundance' | 2 | 45 | 3.156508 | 0.052139 |  |
| Post-hoc contrasts | B | SE | df | t | p |
| pre_peak - pre_trough | X | X | X | X | X |
| pre_peak - sham | X | X | X | X | X |
| pre_trough - sham | X | X | X | X | X |
|  | Df | Df.res | F | p |  |
| 'central_sigma_abundance' | 2 | 45 | 8.475196 | 0.000752 |  |
| Post-hoc contrasts | B | SE | df | t | p |
| pre_peak - pre_trough | 0.015222 | 0.091094 | 45 | 2.732606 | 0.00895 |
| pre_peak - sham | -0.00724 | 0.091094 | 45 | 1.300613 | 0.20001 |
| pre_trough - sham | -0.02247 | 0.091094 | 45 | 4.033219 | 0.00021 |
|  | Df | Df.res | F | p |  |
| occipital_offset' | 2 | 45 | 3.275011 | 0.047004 |  |
| Post-hoc contrasts | B | SE | df | t | p |
| pre_peak - pre_trough | 0.206954 | 6.649754 | 45 | 1.977196 | 0.05417 |
| pre_peak - sham | -0.04383 | 6.649754 | 45 | 0.418718 | 0.67741 |
| pre_trough - sham | -0.25078 | 6.649754 | 45 | 2.395914 | 0.0208 |
|  | Df | Df.res | F | p |  |
| occipital_exponent' | 2 | 45 | 3.068049 | 0.056352 |  |
| Post-hoc contrasts | B | SE | df | t | p |
| pre_peak - pre_trough | X | X | X | X | X |
| pre_peak - sham | X | X | X | X | X |
| pre_trough - sham | X | X | X | X | X |
|  | Df | Df.res | F | p |  |
| occipital_theta_abundance' | 2 | 45 | 10.55111 | 0.000175 |  |
| Post-hoc contrasts | B | SE | df | t | p |
| pre_peak - pre_trough | 0.030612 | 0.144317 | 45 | 3.892157 | 0.00033 |
| pre_peak - sham | -0.00131 | 0.144317 | 45 | 0.166978 | 0.86814 |
| pre_trough - sham | -0.03193 | 0.144317 | 45 | 4.059135 | 0.00019 |
|  | Df | Df.res | F | p |  |
| 'occipital_alpha_abundance' | 2 | 45 | 2.793389 | 0.071854 |  |
| Post-hoc contrasts | B | SE | df | t | p |
| pre_peak - pre_trough | X | X | X | X | X |
| pre_peak - sham | X | X | X | X | X |
| pre_trough - sham | X | X | X | X | X |
|  | Df | Df.res | F | p |  |
| 'occipital_sigma_abundance' | 2 | 45 | 0.888401 | 0.418403 |  |
| Post-hoc contrasts | B | SE | df | t | p |
| pre_peak - pre_trough | X | X | X | X | X |
| pre_peak - sham | X | X | X | X | X |
| pre_trough - sham | X | X | X | X | X |

| **Table F eBOSC statistics from post-stimulation period** | | | | | |
| --- | --- | --- | --- | --- | --- |
|  | Df | Df.res | F | p |  |
| 'frontal_offset' | 2 | 45 | 3.252743 | 0.047927 |  |
| Post-hoc contrasts | B | SE | df | t | p |
| pre_peak - pre_trough | 0.265652 | 6.473472 | 45 | 2.534944 | 0.01479 |
| pre_peak - sham | 0.107229 | 6.473472 | 45 | 1.02322 | 0.31167 |
| pre_trough - sham | -0.15842 | 6.473472 | 45 | 1.511724 | 0.1376 |
|  | Df | Df.res | F | p |  |
| 'frontal_exponent' | 2 | 45 | 3.12432 | 0.053633 |  |
| Post-hoc contrasts | B | SE | df | t | p |
| pre_peak - pre_trough | X | X | X | X | X |
| pre_peak - sham | X | X | X | X | X |
| pre_trough - sham | X | X | X | X | X |
|  | Df | Df.res | F | p |  |
| 'frontal_theta_abundance' | 2 | 45 | 0.013075 | 0.987014 |  |
| Post-hoc contrasts | B | SE | df | t | p |
| pre_peak - pre_trough | X | X | X | X | X |
| pre_peak - sham | X | X | X | X | X |
| pre_trough - sham | X | X | X | X | X |
|  | Df | Df.res | F | p |  |
| 'frontal_alpha_abundance' | 2 | 45 | 0.076834 | 0.926165 |  |
| Post-hoc contrasts | B | SE | df | t | p |
| pre_peak - pre_trough | X | X | X | X | X |
| pre_peak - sham | X | X | X | X | X |
| pre_trough - sham | X | X | X | X | X |
|  | Df | Df.res | F | p |  |
| 'frontal_sigma_abundance' | 2 | 45 | 2.248611 | 0.117277 |  |
| Post-hoc contrasts | B | SE | df | t | p |
| pre_peak - pre_trough | X | X | X | X | X |
| pre_peak - sham | X | X | X | X | X |
| pre_trough - sham | X | X | X | X | X |
|  | Df | Df.res | F | p |  |
| 'central_offset' | 2 | 45 | 2.316259 | 0.110291 |  |
| Post-hoc contrasts | B | SE | df | t | p |
| pre_peak - pre_trough | X | X | X | X | X |
| pre_peak - sham | X | X | X | X | X |
| pre_trough - sham | X | X | X | X | X |
|  | Df | Df.res | F | p |  |
| 'central_exponent' | 2 | 45 | 1.913945 | 0.159313 |  |
| Post-hoc contrasts | B | SE | df | t | p |
| pre_peak - pre_trough | X | X | X | X | X |
| pre_peak - sham | X | X | X | X | X |
| pre_trough - sham | X | X | X | X | X |
|  | Df | Df.res | F | p |  |
| 'central_theta_abundance' | 2 | 45 | 0.730922 | 0.487093 |  |
| Post-hoc contrasts | B | SE | df | t | p |
| pre_peak - pre_trough | X | X | X | X | X |
| pre_peak - sham | X | X | X | X | X |
| pre_trough - sham | X | X | X | X | X |
|  | Df | Df.res | F | p |  |
| 'central_alpha_abundance' | 2 | 45 | 0.442127 | 0.64543 |  |
| Post-hoc contrasts | B | SE | df | t | p |
| pre_peak - pre_trough | X | X | X | X | X |
| pre_peak - sham | X | X | X | X | X |
| pre_trough - sham | X | X | X | X | X |
|  | Df | Df.res | F | p |  |
| 'central_sigma_abundance' | 2 | 45 | 2.266612 | 0.115374 |  |
| Post-hoc contrasts | B | SE | df | t | p |
| pre_peak - pre_trough | X | X | X | X | X |
| pre_peak - sham | X | X | X | X | X |
| pre_trough - sham | X | X | X | X | X |
|  | Df | Df.res | F | p |  |
| occipital_offset' | 2 | 45 | 1.245716 | 0.297467 |  |
| Post-hoc contrasts | B | SE | df | t | p |
| pre_peak - pre_trough | X | X | X | X | X |
| pre_peak - sham | X | X | X | X | X |
| pre_trough - sham | X | X | X | X | X |
|  | Df | Df.res | F | p |  |
| occipital_exponent' | 2 | 45 | 1.285241 | 0.286541 |  |
| Post-hoc contrasts | B | SE | df | t | p |
| pre_peak - pre_trough | X | X | X | X | X |
| pre_peak - sham | X | X | X | X | X |
| pre_trough - sham | X | X | X | X | X |
|  | Df | Df.res | F | p |  |
| occipital_theta_abundance' | 2 | 45 | 0.688778 | 0.507405 |  |
| Post-hoc contrasts | B | SE | df | t | p |
| pre_peak - pre_trough | X | X | X | X | X |
| pre_peak - sham | X | X | X | X | X |
| pre_trough - sham | X | X | X | X | X |
|  | Df | Df.res | F | p |  |
| 'occipital_alpha_abundance' | 2 | 45 | 0.014586 | 0.985524 |  |
| Post-hoc contrasts | B | SE | df | t | p |
| pre_peak - pre_trough | X | X | X | X | X |
| pre_peak - sham | X | X | X | X | X |
| pre_trough - sham | X | X | X | X | X |
|  | Df | Df.res | F | p |  |
| 'occipital_sigma_abundance' | 2 | 45 | 1.281194 | 0.28764 |  |
| Post-hoc contrasts | B | SE | df | t | p |
| pre_peak - pre_trough | X | X | X | X | X |
| pre_peak - sham | X | X | X | X | X |
| pre_trough - sham | X | X | X | X | X |
