# Supplementary material for: A closed-loop auditory stimulation approach selectively modulates alpha oscillations and sleep onset dynamics in humans
Source: PLoS Biol. 2024 Jun 18;22(6):e3002651. doi: 10.1371/journal.pbio.3002651 (PMC11185466; doi:10.1371/journal.pbio.3002651)
Supplement: Attachment — Submitted filename: response_to_reviewers.docx [file pbio.3002651.s002.docx]

**Response to reviewers’ Alpha closed-loop auditory stimulation modulates waking alpha oscillations and sleep onset dynamics in a phase-dependent manner in humans**

We thank the editor for the opportunity to submit a revised version of our manuscript. The reviewer’s comments were fully addressed in this version, including the addition of new data and subsequent analyses. Below you can find a point-by-point response.

We thank the reviewers for the time dedicated to this thorough revision of our manuscript and their insightful suggestions, which have contributed to enhance the quality of the work. We have made every effort to respond to your questions, and to implement your suggested changes.

In addition, we would like to sincerely apologise for our delay in re-submitting. Unfortunately, our university was struck by a catastrophic server failure last year, leading to the unrecoverable loss of majority of the data (11/20 participants) collected for experiment 1. In order to mitigate this loss, we have worked hard to re-collect, re-process, and analyse these data, in addition to making the suggested modifications. Nevertheless, we are happy to report that we have restored the sample, and the effects remain largely the same, which speaks to the reproducibility of the initial findings. We thank you for your patience, whilst we have dealt with this unfortunate situation.

Below, you will find your comments and questions in black, followed by our responses in blue. We have generated a great many new plots to address some of the more complex points, and we hope that these afford some additional clarity.

For simplicity we have highlighted below the pages corresponding to each reviewer’s comments.

Reviewer 1, pages 2 – 24

Reviewer 2, pages 25 – 28

Reviewer 3, pages 29 – 44

References, pages 45 – 47

**Reviewer #1:**

In a series of experiments using closed-loop auditory stimulation on awake adult humans, the authors explore the physiological effects of targeting alpha oscillations as measured at two different electrode sites (Pz, Fz, referenced to mastoid). The topic is interesting and timely, given recent interest in closed loop stimulation, and adds several pieces of information that will be helpful to others wishing to causally manipulate these higher frequency brain oscillations. While not definitive, the results are consistent with a phase-reset model of brain stimulation, and suggest some effects of manipulating alpha-band oscillations.

The paper is extremely dense and must be deciphered, in part owing to its complex nature and in part owing to the structure and presentation of the work.  There are many places in which the work could better convey the results and the authors' ideas more clearly and powerfully, and there are some places in which methods details and particularly justifications for design and analytic choices are needed (see suggestions).

There is some theoretical backing for the work, though the framing is largely exploratory. There is room to better integrate the work with existing literature and ideas about how sound affects brain activity and the nature and roles of alpha oscillations.

We thank the reviewer for the support to our work and the thoughtful suggestions on how to improve the manuscript. We have addressed all questions below and have revised the manuscript accordingly.

**Major comments:**

**1.** The abstract and title ("alpha closed-loop auditory stimulation modulates (...) sleep onset dynamics in a phase-dependent manner in humans" ) as well as various statements of the findings ("we have shown the functional significance of this approach, during the transition to sleep") generates high expectation that the authors were able to successfully influence sleep onset. However, it seems that one of the stimulation conditions just prevented subjects from getting into deeper sleep as compared with the other stimulation condition, or sham.  While this series of studies represents a valuable foray into causally manipulating alpha and trying to observe the physiological responses, the set up seems to detract from these efforts and makes the claim of results seem overstated.

Our results do show phase-dependent differences in the falling asleep process. However, we understand that the expectation might be that we facilitate sleep onset, while the opposite was observed for one of the conditions. To clarify this, we have now changed the wording of statements in the abstract and introduction such that the reader is readily aware of the direction of our findings.

Abstract, lines 13-15:

*“Finally, we demonstrate the functional relevance of our approach by showing that αCLAS can interfere with sleep onset dynamics in a phase-dependent manner.”*

Introduction, lines 93-97:

*“We showed that the dynamics of sleep onset can be modulated in a phase-dependent manner, such that αCLAS administered prior to the troughs of alpha oscillations resulted in ‘shallower’ sleep. This was characterised by a reduction in the amount of non-rapid eye movement (NREM) sleep stages 2/3 (i.e., N2+) and longer latencies for N2+ sleep, accompanied by a decrease in alpha frequency and a limited reduction in alpha activity.”*

**2. Density/readability suggestions:**

a) There are about 5 "experiments" split over 3 "studies". The first encompasses experiments 1 and 2 (labelled as such), the second experiments 3 and 4 (also labelled), and the third study seems to have a fifth experiment (not called experiment 5?). The reader would have an easier time following this structure with a bit of an orientation as to the overall plan and goals and what each investigation looks at in the introduction, as well as consistent use of naming (like using 'experiment 5' for the last one).

We thank the reviewer for these suggestions. To improve clarity, we now only refer to experiments, instead of studies and experiments. We have expanded the Introduction to include clearer information on each experiment, signposting the main aims and findings per experiment.

Introduction, lines 82-97:

*“We first explored αCLAS ability to induce phase-dependent modulations to the alpha rhythm in two cortical locations with comparatively different endogenous alpha power (i.e., frontal – lower, and parietal – higher alpha power, [1]; experiments 1 and 2 respectively). We observed that repetitive-αCLAS induced phase-dependent modulations of alpha power, frequency, and connectivity in a spatially-specific manner. To further understand these effects, we used single-pulse αCLAS (experiments 3 and 4). This approach confirmed that the effects of αCLAS are indeed dependent on the endogenous amplitude of alpha oscillations and can be explained using oscillator theory and a phase-reset model. Finally, we applied these principles during the transition to sleep, a process in which alpha oscillations become naturally dampened (experiment 5). We showed that the dynamics of sleep onset can be modulated in a phase-dependent manner, such that αCLAS administered prior to the troughs of alpha oscillations resulted in ‘shallower’ sleep. This was characterised by a reduction in the amount of non-rapid eye movement (NREM) sleep stages 2/3 (i.e., N2+) and longer latencies for N2+ sleep, accompanied by a decrease in alpha frequency and a limited reduction in alpha power.”*

b) The rationale and meaning of some of the analyses is not always clearly explained when they are at first encountered, which in this methods-at-end format is already in the Results. For example, it was hard to follow why ISI is looked at in theta and sigma bands, and I did not understand why and on what basis the Pz and Fz were selected and followed through the various experiments.

We have performed a substantial revision of the manuscript with a longer explanation of the analyses in the Results section and more in-depth justification of our rationale throughout. We believe that this has improved the readability of the manuscript and thank the reviewer for the specific suggestions.

Regarding the basis in which Pz and Fz were selected. When first exploring whether αCLAS could induce phase-dependent modulations to the alpha rhythm, we decided to focus on two locations that have comparatively different endogenous alpha power during rest, eyes-closed conditions. This also allowed us, later on, to identified a spatial specificity in our findings. We have now added this rationale to the manuscript.

Introduction, lines 82-86:

*“We first explored αCLAS ability to induce phase-dependent modulations to the alpha rhythm in two cortical locations with comparatively different endogenous alpha power during rest eyes-closed conditions (i.e., frontal – lower, and parietal – higher alpha power,* [1]*; experiments 1 and 2 respectively).”*

Results, lines 102-105:

*“We first tested whether αCLAS could successfully deliver phased-locked stimuli to the resting alpha oscillation at different cortical locations. It is well established that during rest with eyes-closed alpha power is typically higher in parietal and occipital areas than frontal regions* [1]*.”*

Results, lines 265-271:

*“To understand why sounds administered at different phases of the alpha oscillation appear to speed it up or slow it down in a spatially-specific manner we conducted two new experiments, similar to the previous ones, but with the critical difference that sounds were administered as isolated/single-pulse stimuli. For these two new experiments we targeted again the alpha rhythm at Fz (where we previously observed an effect of αCLAS on alpha activity; experiment 1) and Pz (where we did not observe an effect of αCLAS; experiment 2), respectively (experiment 3: N=8; experiment 4: N=7).”*

Due to the time requirements needed to run experiment 5 (4 visits per participant) we only focus on one location.

Results, lines 375-378:

*“To investigate this, sixteen participants took part in a within-subjects, sham-controlled, cross-over nap study (experiment 5), in which repetitive-αCLAS was once more employed, phase-locked to alpha at Fz, where we previously demonstrated an effect of αCLAS.”*

Regarding the ISI analysis in experiment 5, where we looked at the ISI in theta and sigma bands. We performed this analysis to understand whether the ISIs remained locked to frequencies in the alpha band or whether the ISIs shifted as the brain changed its state and consequently the predominant frequencies (i.e., alpha during wakefulness, theta during N1 and N2, sigma during N2). If the ISIs had remained locked to the alpha band as participants transition from awake, to N1 and N2 this would suggest that our closed-loop approach was not following brain’s activity. This is an important consideration when using closed-loop experiments that aim to modulate brain activity, while being informed by ongoing brain state. The question posed here is the direction of causality, i.e., if the ISIs change during the course of the experiment, do shorter ISIs speed up the brain’s rhythms or do faster brain rhythms lead to shorter ISIs? This question cannot be answered while the stimulation (i.e., sounds) are being delivered, because these have an impact on brain activity, as demonstrated in experiments 1-4. Thus, in experiment 5 we took advantage of the fact that in the sham condition the αCLAS algorithm was implemented, locked to the same phase as in the ‘pre-peak’ condition, but the sound output was off. This meant that we could follow what the algorithm was doing without inducing any perturbation to the brain. As the ISIs shifted with brain state, this indicates that the ISI is dependent on the brain’s physiology.

To reduce the density of the material presented in the main manuscript, we have now removed the analysis of ISIs per vigilance state from Figure 7 and have instead pointed to the analysis in Figure S14. The legend of Figure S14 now provides further explanation to the importance of this analysis.

Results, lines 397-400:

*“Furthermore, the distribution of ISIs differed between vigilance states further suggesting that the ISI and therefore the closed-loop algorithm used is dependent on the brain’s physiology (see Figure S14).”*

***Figure S14. Inter stimulus intervals (ISIs) per sleep stage.*** *ISIs of the sounds delivered in the pre-peak and pre-trough conditions and dummy sounds in the sham condition (i.e., no sound output) were investigated for each vigilance state (awake – W, N1 and N2 sleep). As participants transitioned from awake to N1 and N2 during experiment 5, the main frequencies detected in the phase-locking electrode also changed (alpha is more predominant during wakefulness, theta during N1 and sigma during N2). The shift in ISIs detected per vigilance stage for each stimulation condition indicates that the αCLAS algorithm followed brain activity.*

*Violin plots show the percentage of ISI’s in each frequency band, in each sleep stage, in each condition. Stats indicate output of linear mixed effects model [ISI_percentage ~ sleep_stage + (1|participant)]. * p <0.05, ** p<0.01, ***p<0.001.*

c) The article body has 8 large compound figures, many of which are made of smaller subplots - some as many as 47 (Figure 8) with a half-page of caption. Some of the plots are quite specialized and are likely to be unfamiliar graphic representations to many readers. I would suggest to select main questions of interest and move some of the other plots to Supplementary material, and/or find some way to more globally summarize the patterns in large numbers of similar subplots.

We agree that the quantity of data and plots in the manuscript became unwieldy, and to improve readability, we have carried out several large changes. Firstly, the sidebar concerning changes to connectivity has now been moved to the supplementary materials, and the extended investigation of its origin has been removed entirely, so as to focus the narrative. This immediately brings down the total number of figures in the manuscript from 8 to 6, and hopefully improves clarity. Secondly, several panels have been moved from the remaining plots (3, 5, and 6) to the supplementary materials to minimise the number of similar subplots.

Specifically:

- Figure 3: circular plots were removed, as we realised the same information is present in figure 4.
- Figure 3: AEP plots from different alpha power octiles have been moved to the supplementary materials, as we consider the main point here (inverse relationship between alpha power and strength of phase-reset) to be summarised in figure 3’s regression plot.
- Figure 5: We have moved details on the inter-stimulus interval to the supplementary materials, as we realised these were either recapitulating findings from experiment 1 or were not directly pertinent to answering our scientific questions.
- Figure 6: This was previously the largest plot, and we have minimised its size and number of subplots by moving information on N1 sleep to the supplementary materials, as this does not form part of our main finding, and by including only plots from frontal EEG metrics in the main manuscript. The remainder exists in the supplementary materials.

d) Related, the figures could use more interpretable names / labels. There is quite some deciphering to be done for example to figure out how Figure 6. A, B, C, i - iv in Experiment 3 at Fz relate to similar plots for Exp 4 at Pz.

We agree with the reviewer that the figure labels were not optimal. We have changed the figure labels to make them clearer, adding i - iv to the experiment 4/Pz side of the figure in addition to the experiment 4/Fz side. We have also increased the size of these labels to aid interpretability. We hope this rows and columns format will now be fairly intuitive.

**3.** As the authors acknowledge, auditory evoked responses occur in response to sound and have a similar topography. There is considerable literature on the existence of an entrainment mechanism of auditory networks close to this frequency band (generally very slightly lower, like 4-9 Hz, though without hard boundaries). It was unclear to me to what degree the authors were able to separate an alpha modulation from a passing an AEP that might be composed of a normal evoked response and some addition from ongoing alpha oscillations through the band-pass filter, and how phase-resetting might be separated from a process of progressive entrainment. Is there some way of distinguishing these possibilities in these data?

We thank the reviewer for the critical questions posed here. The first question focuses on: **(1)** what is evoked by the repetitive sounds and, hence, **(2)** to what are proceeding sounds phase-locked to, i.e., a passing AEP or an ongoing alpha oscillation. The second question focuses on separating phase-reset from progressive entrainment.

Answer to question 1: The distinction between the two cases is exceptionally difficult. Whatever the source of the effect (genuine phase-reset of oscillations or simple addition/superposition of potentials), it is temporally coupled to ongoing alpha band activity in the case of alpha phase-locked stimulation. This means it will appear to be part of alpha activity, whether it shares an origin or not. This is especially problematic in a measure like EEG, in which electrophysiological features can have independent sources and also appear superimposed in space at the scalp, making them extremely difficult (if not impossible) to separate. This issue is compacted further by the notion of cortical excitability, which supposes that the brain will be more or less excitable depending on the phase of ongoing oscillations.

Let’s consider the case of the additive model. If the brain is most excitable at the peak of an oscillation, then in simple terms a larger evoked potential may be elicited at this phase. However, the positive deflection of that potential may be perfectly aligned with the trough of the ongoing alpha, making it appear smaller in magnitude. In the opposite example, the trough is the least excitable but the potential is added to the already positive peak of a subsequent oscillation, making it appear larger in magnitude.

In the case of a pure phase-reset one would not have to worry with the phase of the endogenous oscillation assuming that every and all oscillators contributing to alpha activity are reset in precisely the same manner. This seems very unlikely, since we measure innumerable oscillators from many different sources, and may not reset all of them. This leaves us with a situation in which some oscillators are reset and some continue, another situation in which an additive and phase-reset model may not be distinguishable in the EEG. Indeed, the inability to adjudicate between which model best describes the generation of an evoked response from EEG data has been discussed at length [2–16] Current evidence suggests that the differentiation between these models might not be feasible if based solely on macroscopic EEG recordings, indicating that invasive recordings are needed to disentangle this question [4,17].

Despite the clear difficulties in disentangling oscillations from evoked potential we have made our best attempt to describe what might be occurring in our closed-loop experiment. However, it must be said, that the issue remains somewhat ambiguous.

In order to address **(1)**, i.e., to show the immediate response in the EEG to repetitive αCLAS we plotted ERP’s for each of the first 10 stimuli in experiment 1 where we used a block design with repetitive αCLAS. Below is shown, for each stimulus, the evoked potential at Fz (from the αCLAS EEG system) for each phase condition (coloured lines), the average of all four conditions (black line), the stimulus onset (vertical grey line) and statistically significant differences from zero across all subjects and conditions (red scatter points, significantly greater than zero, blue scatter points, significantly less than zero). What is clearly shown is that the first stimulus evokes a, broadly, conventional response to a sound, with high frequency components followed by a low frequency component (i.e., a stereotypical AEP). This is especially clear when averaging across conditions, and it is also evident that the low frequency component of this evoked potential moves steadily closer to stimulus onset with each subsequent stimulus, demonstrating that it is only the first stimulus which evokes this low frequency response. Note: the frequency of stimulation is according to the alpha frequency, i.e., there is a stimulus ~ every 100 ms, and these plots are describing around 1 second of stimulation (i.e., ~10 stimuli). Once this low-frequency component has passed the predominant response in the EEG is a short-lived higher frequency component.

While the figure above was produced using the broadband signal (2-30 Hz), the specificity of this low frequency potential to the first stimulus can be seen yet more clearly when filtering the EEG using a 5 Hz lowpass filter, see below:

For consistency with our other analyses, and considering our interest in the alpha band, we also plotted the same data filtered 7.5-12.5 Hz. As with previous analyses, it can be seen that conditions are orthogonal before a stimulus and more aligned after a stimulus. An oscillation post-stimulus, greater in magnitude than pre-stimulus, can be seen in both the average trace (black line) and the timepoints at which the group was greater or less than zero (red and blue scatter points, respectively).

This effect can be seen more clearly still, when applying a Hilbert transform to the alpha-filtered signal to obtain phase (coloured lines) and conducting a Rayleigh test to identify periods of statistically significant phase homogeneity between conditions (black scatter points):

Whilst these provide good evidence for **(1)**, answering **(2)** is not so straightforward. After all, a reset oscillation and an evoked potential are indistinguishable by EEG at some point. However, we surmise that if we were simply passing repeated evoked potentials through our closed-loop then the stimulus would be highly periodic, i.e. the sound evokes a potential, which triggers a sound, which evokes a potential. This takes us to question 2.

Answer to question 2: Above we discussed the phase-reset model and now we focus on the question of entrainment. Entrainment is typically defined as synchronization in phase or amplitude of neural oscillatory activity to periodic stimulation. Based on this definition, stimulus periodicity is a requisite. To test whether our closed-loop approach delivered stimuli in a periodic fashion we tested the autocorrelation of the inter stimulus interval across 20 lags, in which a value of 1 would indicate a perfectly periodic stimulus and lower values indicate a deviation from periodicity (see figure below). The 4 different colours in the figure represent the four phase conditions and the error bars indicate standard error of the mean (ISIs retrieved from the blocks in experiment 1). It is clear that the stimulation we administered is far from periodic, with the autocorrelation coefficient quickly dropping to <.1 after only 2 lags. We propose that if the sound was simply entraining itself, via the EEG, then we would see a highly periodic stimulus. This is not the case here (see Figure below, which has now been added as Figure S6).

In addition, the reviewer mentions “*the existence of an entrainment mechanism of auditory networks close to this frequency band (generally very slightly lower, like 4-9 Hz, though without hard boundaries)*”. We believe the reviewer is alluding to the phenomenon known as envelope tracking, where the phase of low-frequency neural activity measured in the EEG/MEG correlates with the amplitude envelope of speech. Indeed, while natural speech does not have a robust temporal rhythmicity [18], the idea that neural activity tracks the envelop of speech is not without debate. Currently, two main competing models have been put forward in this debate: entrainment (i.e., phase alignment) and transient neural responses [19–23]. Here, again, the jury is still out to whether entrainment is present when speech is not rhythmic.

We have now included information in the manuscript and supplementary files according to the analyses we presented above and have included additional information on the current literature discussion on the difficulty to adjudicate between models of the origins of evoked potentials.

Results, lines 225-227:

*“The effects of αCLAS on power and frequency cannot be simply explained by entrainment to a periodic stimulus, as the stimuli delivered by αCLAS was aperiodic (****Figure S6****).”*

Discussion, lines 514-519:

*“There is no unanimous consensus on how related or unrelated evoked potentials are to phase reset of oscillations* [24] *with ongoing attempts to adjudicate between the best models to explain the generation of evoked responses from EEG data*[2–16]. *Current evidence suggests that the differentiation between these models might not be feasible if based solely on non-invasive scalp EEG recordings*[4,17]*”*

**4.** Why are the phase locking-values in Exp 2 and particularly Exp 1 so much lower using the hd-EEG system than with the ecHT device? (e.g., mean for Exp 1: 0.84 vs. 0.54) It is explained that the difference is because of the differences in EEG montages; however, evidence does not seem to be presented. Can the authors confirm by running the analysis on a re-referenced version of whichever set of electrodes is closest to Mastoid vs. Fz in their cap?

We thank the reviewer for this suggestion. Below is the phase-locking accuracy (resultant) at Fz (taken from the hd-EEG system) in experiment 1, contrasting the Laplacian reference with the right mastoid reference (TP10 also from the hd-EEG system). There is a subplot for each phase condition. Statistics show the result of a paired t-test comparing the two referencing schemes, *** p <.001. The phase-locking accuracy is higher when referencing to the right mastoid, as expected considering the reference used for the ecHT device.

We have now added this information in the revised manuscript and supplementary material.

Results, lines 136-140:

*“The lower resultant vector lengths displayed for the hd-EEG are a consequence of the scalp current density (i.e., Laplacian) reference used here (see Figure S1 for a comparison between resultants at the target electrode in the hd-EEG system using Laplacian or mastoid reference as in the αCLAS EEG system).”*

***Figure S1. Comparison of the resultant values using different referencing schemes.*** *The resultant was calculated in electrode Fz of the hd-EEG system in Experiment 1 using two referencing schemes, Laplacian (as used for all analysis in the main manuscript) and right mastoid (to match the referencing scheme used in the αCLAS EEG system). Channel Fz in the hd-EEG system was adjacent to this channel in the αCLAS EEG system, and the mastoid channel in the hd-EEG system was taken from TP10. The resultant values using the right mastoid reference are higher and more similar to those reported for the αCLAS EEG system.*

*Violin plots show the resultant for each targeted phase in Experiment 1 in each reference scheme. Black lines represent each participant. Stats indicate output of paired samples t-test between the resultant for each reference scheme, ***p<0.001.*

**5.** Nap studies usually occur in the afternoon, for reasons relating to circadian rhythm and propensity to sleep. Why was Study 3 conducted in the morning, when subjects are most likely farthest from sleep? (Related, why were normal, healthy, well-rested subjects falling asleep in a few minutes with their eyes closed in the morning while loud clicks were played in Study 3?)

Sleep propensity shows a well-established pattern across the day with shortest latencies in the afternoon and longer latencies in the morning. Most naps studies aim to study sleep, not the changes involved in the transition between wakefulness and sleep and are therefore usually conducted in the afternoon, as the reviewer points out. Here we are interested in effects on sleep latency and brain features associated to falling asleep. Thus, we chose a time of day when sleep latencies are longer.

In young participants, sleep latencies during the day are indeed surprisingly short. In a larger study in which we compared the diurnal pattern of sleep latencies across three age groups, including young adults (20–30 years old) we found that in the morning sleep latencies at 9 and 11 am were around 10 minutes in these young participants (n=41). Young participants are sleepy during the day even when they are in bed for 8 hours during the night preceding the sleep latency tests [25].

We have now added this information in the Methods section, lines 662-665:

*“Visits were scheduled in the morning as sleep latencies vary during the day, with longer latencies observed in the morning compared to afternoon in young adults* [25]*. Thus, providing a possible longer window in which to study the effects of αCLAS on the dynamics of falling asleep.”*

**6.** More details and clarifications are needed as regards methods and justifications for design choices. For example, what was the justification for 80 dB (presumably SPL) sound presentation? This is considered quite loud for auditory experiments. How were the stimulation phases selected? (pre-peak 330 decrees, post-peak 60 degrees, etc.)

In auditory stimulation studies concerning sleep there is a delicate balance to be struck when it comes to volume; too great and the participant will be awakened, too little and the brain (in an already less responsive state) may show no/minimal response. We found that during a previous pilot study, participants became drowsy to the point of near sleep-onset within 8 minutes of 80 dB sound stimulation, in a setting which was not especially optimised for sleep. Hence, we considered this volume appropriate for our nap study. Moreover, we wanted to maintain as much consistency, as possible, across different experiments. Finally, the participants did indeed sleep, suggesting that the volume was not a hindrance.

Regarding the phases chosen, we simply chose four phases which were maximally separated by 90 degrees, i.e., orthogonal. These phases were not selected on any particular basis, since the aim of our experiments was simply to identify if there was an effect of instantaneous alpha phase on the response of the brain to sound.

For clarity, we have added the following information to the Methods section, lines 683-692:

*“Across experiments, sounds were 80 dB, 20 ms pulses of pink noise, delivered to the participants via earphones (Etymotic model ER-2 in experiments 1-4 and Shure model SE215-CL-EFS in experiment 5). During pilot testing with continuous stimulation over a period of minutes the volume of the sound did not prevent drowsiness and was therefore also deemed appropriate for experiment 5. Sounds were phase-locked to 4 orthogonal phases (i.e., providing maximum separation between each phase condition) of alpha in experiments 1-4 (60°, 150°, 240° and 330°) and two phases in experiment 5 (150° and 330°). In the sham condition in experiment 5, phase-locking was still computed, and markers were logged for all ‘stimuli’ (dummy targeting 330°), but the volume was set to 0 dB ensuring that no sounds were played to the participant.*

**7.** More information is needed as regards sleep scoring - which system was used? Was the scorer blinded? (someone is acknowledged for scoring them and there is some discussion of finer-grained measures)

Sleep was scored according to the AASM criteria, in which 30-s epochs are labelled as wake, NREM1, NREM2, NREM3, or REM, according to various biological signatures (alpha oscillations, K-complexes, spindles, slow oscillations, muscle atonia, eye-movements, etc). This scoring was carried out by a scorer who was blind to the condition and was unfamiliar with the details of the experimental protocol.

We have now added more information regarding sleep scoring to the Methods section, lines 823-826:

*“Sleep was scored by an experienced sleep technician blinded to the experimental condition and unfamiliar with the details of the experimental protocol, according to AASM guidelines* [26] *Scoring provided 30 s epochs labelled as wake, non-rapid eye movement (NREM or N) N1, N2, N3 or rapid-eye movement (REM) sleep.”*

The other ‘finer-grained’ measures, i.e., offset, exponent and abundance in theta, alpha and sigma bands was obtained by the eBOSC toolbox as described in the manuscript.

We have added more information to the Methods section, lines 837-847:

*“We employed eBOSC* [27] *to estimate both rhythmic, i.e. oscillatory, and arrhythmic, i.e. aperiodic or 1/f, EEG activity (i.e., offset and exponent). For eBOSC analysis, for each channel, EEG data were divided into 30 s epochs, since, during a dynamic process such as sleep onset, a single fit and threshold for oscillation detection is unlikely to be optimal. For each of these epochs, eBOSC was run between 1-30 Hz, in steps of 0.25 Hz, excluded frequencies for background 1/f fit 7:16 Hz. For rhythmic activity, only oscillations with a duration of at least 3 cycles were analysed to estimate abundance (i.e., the duration of the rhythmic episode relative to the length of the analysed segment). The abundance computed was then averaged within canonical bands for each epoch. This was done for all channels (F3, F4, C3, C4, O1, O2) before further averaging into regions (frontal: F3, F4; central: C3, C4; occipital: O1, O2).”*

**8.** Why PLV and PLI? If PLI zero phase was discarded, does that mean PLV is potentially contaminated by volume conduction artifacts? Is that was is referred to when the authors describe relative sensitivity to type 1 and type 2 errors? Note that the meanings of these connectivity metrics is not referred to in the discussion; it is not clear what these analyses (and having included two measures) adds.

Indeed, the drawback of PLV is that immediate volume conduction may influence results. Meanwhile, the drawback of PLI is that it discards genuine instantaneous connectivity. This is why we used both measures.

However, in light of the comments regarding the extensiveness of the manuscript and the request to make it more focused we have opted for moving the basic connectivity analyses (i.e., showing the effect of stimulation phase on connectivity) to the supplementary materials, and removing the extended analyses of their origin entirely. While we find it noteworthy that the phase of stimulation produces different patterns of connectivity, and think it is worth mentioning here in a reduced capacity, we believe that the details might be better explored in a different publication.

**9.** Have the authors considered how changes in strength of the oscillations at different regions may have impacted the connectivity metrics (and their conclusions)?

Whilst we agree that this is especially concerning when it comes to connectivity measures which depend on power/amplitude, the measures we have implemented are based on the phase and are therefore less biased by this issue.

Moreover, the overall strength of oscillations in the alpha band did *not* differ between conditions. Instead, their frequency did, resulting in the changes in power observed *within* the alpha band for experiment 1 (Figure 2 in the manuscript). To better illustrate this, we have computed the change in total alpha power (7.5-12.5 Hz) for experiments 1 and 2. The figure below shows the topography of the ANOVA investigating alpha power change between ‘on’ and ‘off’ periods for the four phase conditions (panels A) and the same for frontal (panels B), parietal (panels C) and all channels (panels D). None of these showed a main effect of change of alpha power, in either experiment 1 or 2.

Given that our connectivity differences between conditions were observed in the overall alpha band, we do not believe this was driving the connectivity results.

As we opted to remove the connectivity results from the main manuscript (as per response above), we have not added this additional analysis to the manuscript.

**10.** How were sample sizes decided upon, which differ quite a bit between the experiments? (Justification/power analysis)

No statistical methods were used to predetermine sample sizes, but our sample sizes are similar to those reported in previous publications investigating the effects of sound stimulation on physiological responses. In particular, for experiments 1, 2 and 5 we defined our sample size based on previous studies using closed-loop auditory stimulation to modulate physiological brain responses. Such studies have been conducted mostly during sleep focusing on targeting slow wave oscillations or NREM sleep features. A recent review of the literature, which included 23 studies on the effects of auditory stimulation during sleep, indicates that sample sizes vary between 5 and 90 participants, mean ± std = 19.4 ± 16.8, median = 15.5 and mode = 13 ([28] data retrieved from Supplementary Table 2 of this publication). Our final sample sizes for experiments 1, 2 and 5 (N=23, N=28 and N=16, respectively) are all in agreement with those typically used in other closed-loop auditory stimulation experiments. Sample sizes for experiments 3 and 4 were based on the fact that auditory evoked potentials are readably identified in individual subjects and that a large number of trials can be obtained within the duration of a typical experimental session (~2 h). We were conservative in acquiring a large number of trials (N=192 trials) per phase condition and use a sample similar to other studies, N=7-10 [13,14,29,30].

We have now clarified this in the Methods, lines 616-621:

*“No statistical methods were used to predetermine sample sizes, but our sample sizes are similar to those reported in previous publications investigating the effects of sound stimulation on physiological responses, which typically vary between 7-10 participants for studies measuring evoked responses as in experiments 3 and 4* [12,13,29,30] *to about 19 for studies using closed-loop auditory stimulation, typically targeting slow-wave sleep activity* [28]*”*

**11.** Why did the authors decide to analyze connectivity in sensor space rather than source space (i.e., brain), which might have been more informative and is quite possible using hd-EEG?

The primary objective with source space analysis is to make more accurate neuroanatomical inferences from M/EEG signal. This was not the objective of our connectivity analyses, where we ‘simply’ wanted to investigate whether an effect of phase on connectivity was present and the general pattern this effect might have.

We appreciate that connectivity at the sensor level comes with a set of assumptions, but would argue that source reconstruction adds yet more assumptions. Source reconstruction requires complex pipelines broadly divided into addressing forward and inverse problems. The forward problem is the easiest to solve, provided that one has realistic and subject-specific head models obtained from MRI data. As part of this study, we did not acquire MRI scans from our participants and therefore cannot build individual head models. The inverse problem is then addressed by generating scalp distributions from electrical potential differences based on a specific algorithm. There are, however, a plethora of inversion algorithms available, persistent controversies regarding the choice of algorithm, with many studies suggesting that more than one algorithm should be compared to establish the reliability of the results (e.g., [31,32]). We also consider that our closed-loop approach may present some unique obstacles for source reconstruction, since it is enforcing a strong correlation between the auditory-evoked activity and the spontaneous activity, and source reconstruction seeks to separate sources on the basis of their correlation.

A recent study comparing functional connectivity estimates from sensor and source spaces concluded that there is a strong correlation for the global connectivity between scalp- and source-level [31]. In contrast, network topology (not investigated in our study) was only weakly correlated, albeit more accurate for functional connectivity metrics that limit the effects of volume conduction/signal leakage, such as PLI. This is also why we employed both PLV and PLI in our analysis of connectivity.

**12.** This paper format has results before methods. Keeping in mind that the reader does not yet have in mind the Methods details at time of reading the results, it would help to have a few more methods details included in the results, for example that there is to be post-peak and post-trough stimulation in Study 3, what electrode or electrodes are considered in the hd-EEG analysis, what the time window is for connectivity analysis following stimulation was, or what the analytic basis for statements like "phase-locking was highly accurate across experiments, greatest around locations targeted" are (in brief). What are the "locations targeted" here? Does this mean the scalp locations? Does it mean Fz and Pz separately? Why both?

We agree that we were very economic with our description of methodology in the Results section which resulted from a combination of the number of analyses and results presented and the word limits.

We have now included more information on key methodological aspects in the main manuscript and figure legends.

Regarding the specific sentence mentioned by the reviewer ("phase-locking was highly accurate across experiments, greatest around locations targeted"), we have modified to be clearer that we are talking about the location of the target electrodes.

Results, lines 182-184:

*“Having shown that αCLAS delivers sounds accurately at the prescribed phase in the two targeted electrode locations, we next examined whether αCLAS induced phase-dependent changes to the alpha oscillations we set out to target.”*

**13.** The explanation for using Fz vs Pz could use more explanation, and a stronger connection to extent literature on alpha origins and roles. The methods section says only "frontal and parietal regions of interest (ROI) were defined a priori" - but how/why/based on what?

Please see answer to the reviewer’s question 2b above regarding the choice of Fz and Pz and the corresponding changes in the manuscript.

Regarding the frontal and parietal ROIs we selected channels surrounding the ‘equivalent' phase-locking channel in the hd-EEG system. The inclusion of 4 channels per ROI is a compromise between spatial specificity (i.e., a small number of channels located close to the phase-locking channel) and sensitivity (i.e., the benefit of having data from more channels to improve signal-to-noise ratio to detect and effect). Another approach would have been to select the channels based on the whole-brain ANOVA, but we had not made previous assumptions about the spatial specificity of the effects, and these would inherently depend on the type of variable being analysed (e.g., power, frequency, etc.) and the target electrode in question (i.e., Fz vs Pz), most likely resulting in an unbalanced number of electrodes per analysis.

We have now added additional information in the Methods, lines 755-760 regarding the justification for the choice of ROIs:

*“ROIs only included electrodes from the hd-EEG system and chosen to balance spatial specificity (i.e., a small number of channels located close to the phase-locking channel) and sensitivity (i.e., the benefit of having data from more channels to improve signal-to-noise ratio to detect and effect), while keeping the number of channels per ROI constant across analyses and experiments.”*

**14.** In Exp 1, targeting Fz, an alpha-CLAS phase effect on power of alpha oscillations was found; this was not found for Pz, although a modulation of alpha power was found; the authors conclude that the effects of alpha-CLAS on alpha power and frequency are location-specific - what does this mean in terms of underlying anatomy?

There are (at least) two concurrent contributory factors to the effect of alpha-CLAS which must be considered. As the reviewer rightly mentions, the anatomy may play a role. For example, auditory evoked potentials are most prominent in frontal/central regions. Simultaneously, as we show in our analysis, the power or amplitude of ongoing oscillations also plays a role, with stronger oscillations more difficult to perturb, and this also differs by brain region, with more prominent alpha oscillations in a parietal region that a frontal one. These two things may in fact very well be related, but the purpose of this work was more focused on investigating the feasibility, mechanisms, and restrictions of this new neuromodulation method, and less to do with making statements on the workings of the brain itself, although the two are clearly related. We chose Fz and Pz on the basis of the factors mentioned above, in the EEG signal these channels are clearly distinct in both their oscillatory activity and their anatomical sources. Again, we simply wanted to understand the scope of our neuromodulatory approach for influencing EEG activity. As such, these sites were not chosen on the basis of any particular brain region, e.g., frontal cortex, auditory cortex, visual cortex, and so on.

We have now added additional information regarding the choice of Fz and Pz (please see answer to the reviewer’s question 2b above).

**15.** How far were the phases of Fz from those stimulated on Pz? What is the phase offset between these two scalp locations, and is it predictable or random?

This is an interesting question, because if the phase offset was predictable and consistently different in a particular phase targeting Fz might have specific effects at Pz. Below is the phase offset between Fz and Pz, at stimulus onset, following Laplacian reference, across all stimuli, across all 4 conditions, in experiment 1. Each line represents an individual participant, the angle represents the average phase offset, and the length of these lines represents the resultant. Whilst the majority of participants exhibit a roughly opposite phase between Fz and Pz, the resultant is quite low (<.4 for most participants) and the spread is not especially focused. The low resultant indicates that the phase offset is not predictable.

**16.** Frequency differences - pg 10 - missing from this analysis is some indication of the degree to which a clear alpha peak is observed in each of these channels. This is important because theta-band activity is found just below the alpha band range, and is often not clearly distinguished from it. Sigma band activity related to spindles also only becomes clear and 'peaky' in N2. Theta is also stronger in frontal midline regions. There should be some discussion of how clearly the authors' analysis is getting at alpha as opposed to theta and sigma.

This is an important point. The presence, and necessity, of genuine alpha oscillations does not feature prominently in the discussion or interpretation of this work. It is, of course, correct that alpha oscillations do not prominently feature in NREM sleep; which is defined to a large extent by their absence. Considering our stimulation had a similar effect on “alpha frequency” across the stimulation period, in spite of reduced alpha oscillations (i.e., participants fell asleep), we may tentatively conclude that our approach and its outcomes are not explicitly and specifically dependent on the presence of prominent alpha oscillations.

The Discussion of the manuscript already contains information on the limitations of our algorithm, which did not include a threshold for “oscillatory” activity or power in a particular band. We have now included added information regarding the observation that the effects on frequency were not dependent on the presence of alpha oscillations, lines 568-572:

*“Future studies can implement closed-loop strategies that include a detection threshold, thus limiting stimulation to periods when the target rhythm is most prominent. Albeit the presence of a genuine alpha oscillation does not seem to be a requisite to induce changes in alpha frequency, as observed in experiment 5 where alpha oscillations are naturally reduced as participants fell asleep.”*

**17.** Is R2 = 0.97 in Fig 4 correct? Please explain in "Statistical Analyses" how this measure is computed here, as there are several methods (see <https://eur02.safelinks.protection.outlook.com/?url=https%3A%2F%2Fjonlefcheck.net%2F2013%2F03%2F13%2Fr2-for-linear-mixed-effects-models%2F&data=05%7C01%7Ch.hebron%40surrey.ac.uk%7Cffa7746eb06c4a19f1d608db77323d9f%7C6b902693107440aa9e21d89446a2ebb5%7C0%7C0%7C638234829891052156%7CUnknown%7CTWFpbGZsb3d8eyJWIjoiMC4wLjAwMDAiLCJQIjoiV2luMzIiLCJBTiI6Ik1haWwiLCJXVCI6Mn0%3D%7C3000%7C%7C%7C&sdata=37clbEaBWIicWf9mFOhUi5WfXUVwDknZaSSFPycCzBM%3D&reserved=0>), and (briefly) how it should be interpreted

We agree that this value appears surprisingly high, although the precise results of linear mixed-effects models can be difficult to predict based on observation of the data alone. At any rate, this particular analysis will be explored elsewhere and no longer features in the manuscript or supplementary information.

**18.** Why are different referencing strategies used across the studies and what are the potential effects on the reported results?

The usage of different references was in part constrained by the different EEG systems used. The αCLAS EEG system used only contains three electrodes, the phase-locking electrode, a reference and ground (as described in the manuscript). Thus, the reference for this system was kept constant, i.e., right mastoid and no re-referencing is possible. For experiments 1-4 we made use of the fact that we acquired in parallel hd-EEG data and used a Laplacian reference for more accurate spatial topographies of the EEG signal. In experiment 5, we used the standard EEG system used for polysomnography (Somnomedics HD) which was referenced to Cz during data acquisition, and we did not perform any referencing with the exception of sleep scoring. Sleep scoring was performed by an external person to the study and they re-referenced the data to the linked mastoids.

As suggested by the reviewer, we compared the resultant between the hd-EEG and αCLAS EEG systems in experiment 1 referenced both to the right mastoid (see answer to question 4 and Figure S1).

In addition, we clarified in the Methods the use of reference across the manuscript, lines 730-733:

*“The hd-EEG data from experiments 1-4 was subjected to a scalp current density (i.e., Laplacian) re-referencing, whilst the EEG data from experiment 5 remained referenced to Cz for all analyses except sleep scoring which was performed with data referenced to the linked mastoids.”*

**19.** Fig 4A: the red to blue color scale is usually used to indicate a divergent scale where the two colors have opposite meanings. Here, blue is closer to 0, red is farther away, and white is at some arbitrary point in between (0.2). I suggest to use a continuous scale for easier interpretation.

We understand the issue and have rectified this with the use of continuous colour maps.

**20.** In some places, it is hard to follow the logic in the Results section; for example, the relative meanings of the "frequency difference" and "change in frequency difference" analyses presented in Figure 4 were not clear.

We agree that this particular analysis was confusing amongst an already crowded manuscript and distracted from the focus. This will be explored elsewhere and hence we have removed it from the submission.

**21.** If alpha phase-dependent auditory stimulation influences the effect of sound on the brain through a process of inhibition (or put another way, relative cortical excitability), why did the authors not explicitly investigate the amplitude of cortical evoked auditory responses by alpha phase? Would the absence of an amplitude difference in the AEPs count against this theory?

While it is tempting to put this theory to test using cortical EEG data, we have highlighted in our previous answer to question 3 that this is not straightforward and that current evidence suggests that invasive recordings are needed to address it [4,17]. Based on this and ongoing discussions in the field regarding the mechanisms of generation of action potentials we believe that this analysis, at this stage and with the data we have, would not help to clarify this issue.

**22.** Some of the conclusions could use some elaboration for clarity, e.g., "Indeed, our results demonstrate that a parietal phase-reset could only be observed in instances of low alpha power (...) This is also in line with oscillator theory, which proposes a phase reset is dependent on the magnitude of both the oscillator and the perturbation, and agrees with prominent theories that the phasic inhibition exerted by alpha oscillations is proportional to their amplitude" - this statement does not explain by lower alpha power in particular should lead to greater susceptibility to resetting. Or, "Secondly, the differential in the magnitude of AEPs at frontal and parietal regions could be a contributory factor to regional differences." - please clarify the proposed link between AEP topography and phase-resetting susceptibility.

Because alpha power is naturally lower in frontal compared to parietal regions and the magnitude of the phase reset is dependent on the amplitude of the oscillator, a perturbation of the same magnitude (i.e., same sound amplitude) is more likely to induce a phase reset when alpha power is lower, i.e., in frontal regions.

Regarding the link between AEP topography and phase-resetting susceptibility. Here we enter again on the discussions regarding how related (or not) the generation of evoked potentials is to the phase reset of oscillations (as in question 3). This is something that we cannot establish and therefore can only point the reader to the possibility that this mechanism might play a role.

To improve the clarity of our discussion we have added the following text to lines 529-537:

*“This is also in line with oscillator theory, which proposes a phase reset is dependent on the magnitude of both the oscillator and the perturbation (in our case the magnitude of the perturbation, i.e., sound volume, is kept constant between experiments, but the oscillator amplitude varies from frontal to parietal regions), and agrees with prominent theories that the phasic inhibition exerted by alpha oscillations is proportional to their amplitude* [33,34]*. Secondly, the differential in the magnitude of AEPs at frontal and parietal regions could be a contributory factor to regional differences, depending on how related the generation of evoked potentials is to the phase reset of oscillations which is currently unresolved* [2–16]*.”*

**Minor:** 
**23.** Some of the colour schemes are not accessible to people with imperfect colour vision, and may be challenging even for people with normal colour vision, e.g., the thin blue, green and grey lines in Fig 8

Regarding, the colour schemes, we have opted for the same colouring scheme throughout the submission, so as to provide consistency across the experiments. Regarding figure 6 (previously figure 8), we have removed a number of panels and increased the thickness of the lines, which we hope will make it more accessible.

**24.** typo page 18 "pre-through" -> "pre-trough"

corrected

**25.** page 20 - no comma after "Despite"

corrected

**26.** Discussion: "We put forward two complementary explanations for this phenomenon supported by our findings." - if would be better to word this statement so it's clearer the explanations are not a priori in this context; the findings would be congruent with them but not supporting them

We have changed this statement to: *“We considered two complementary explanations for this phenomenon which are congruent with our findings.”*

**27.** use of the term "abundance" is unclear (e.g., "differences in frontal and central sigma abundance")

The term stems from the eBOSC toolbox and refers to the total duration of rhythmic episodes relative to the length of the analysed segment. We have now added this definition to the Methods: *“For rhythmic activity, only oscillations with a duration of at least 3 cycles were analysed to estimate abundance (i.e., the total duration of rhythmic episodes as a proportion of the length of the analysed segment).”*

**28.** Statistical Analysis explanation - please clarify what the LMEM significant main effect means (if I am not mistaken, you must compare the fits of two models; the LMEM analysis itself does not provide a directly interpretable p-value)

To obtain F and p values we employed analysis of variance for linear mixed effects models using the MATLAB *anova* function. We have now added this information to the Methods: *“Where these LMEM registered a significant main effect (p ≤ 0.05, as computed using the anova function)”.*

**29.** The freely available github repo seems to require a University of Surrey login (reviewer did not try with personal account)

Apologies, we have made the repo public and have added the link to the manuscript.

**Reviewer #2**:

In this study Hebron and colleagues introduce a closed loop approach using sounds to target human alpha oscillations. They convincingly show in three studies that closed loop stimulation impact alpha power, frequency and connectivity. They also show that closed loop stimulation of alpha modulates sleep onset dynamics in an phase-dependent manner. Overall, I think this is a very timely and substantial work. I just have some additional comments:

We thank the reviewer for this very positive evaluation of our work and its timeliness. We have addressed all comments bellow.

**Major comments:**

1. With regards to study 3: why did the authors focus on pre-peak and pre-trough instead of post-peak and post-trough. Maybe I missed the point, but as judged from study 1 (Figure 2), the post-conditions had a much stronger impact on alpha power. Wouldn't that make them more suitable as targets in order to alter sleep (onset)?

This may well have been the case. Unfortunately, there was considerable overlap between collection and analyses of data across these studies due to time constraints, and hence by the time we had a good grasp of the outcome of alpha-CLAS, the sleep onset study was already well underway. In retrospect, we would be similarly interested to know the effect of these different conditions on sleep onset, but we are sufficiently convinced that the conditions included demonstrate a phase-dependent effect of sound on the sleep onset process.

We have added the following limitation to the discussion, lines 575-580:

*“Additionally, as data for some experiments were acquired in parallel, the two phases targeted in experiment 5 may not have been optimal to differentially affect the sleep onset process, owing to the similarities we observed between these conditions in experiment 1. Nevertheless, the relative consistency between findings across independent samples and experimental designs speaks to the general robustness of the findings and the presence of a phase-dependent effect.”*

In addition, we have made it clearer in the Methods section that data collection for different experiments occurred in parallel, line 627

*“Data collection for different experiments occurred in parallel.”*

**2.** Related to above: was there any impact of alpha stimulation in study 3 on power? And if not how would the authors reconcile this lack of effect?

We thank for the reviewer for highlighting the discrepancy in our analyses across experiments. It is true that the paradigms used in experiments 1 and 5 are highly similar, and we should expect comparable outcomes. Albeit, with some key differences: (1) as people fall asleep the state of the EEG changes dramatically, and this change may have a larger effect on power than that of our stimulation, and (2) we only have low density EEG available in experiment 5, which makes a direct comparison difficult. Despite these potential issues, we have conducted the relevant analysis on the mastoid-referenced Fz electrode (from the αCLAS EEG system) in experiment 5 (previously referred to as study 3), computing power over the first five minutes of stimulation, and log normalising to the 1 minute baseline. Below, we show the time-frequency representation of t-values comparing our two stimulation conditions, which roughly replicate the results of experiment 1. Black lines outline statistically significant clusters.

In addition, below are the Fz data log-transformed to baseline, and averaged across the first 5 minutes of stimulation. Blue line is pre-peak and green line is pre-trough phase condition. Error bars represent the standard error of the mean. Blue and green scatter points indicate statistically significant differences from zero, as per one sample t-tests, in pre-peak (blue) and pre-trough (green) conditions respectively. Black scatter points indicate statistically significant differences between pre-peak and pre-trough stimulation conditions, as per paired t-test.

Overall, we observe that there is greater power at around 8 Hz in pre-trough stimulation compared to the pre-peak condition.

We have now added this analysis to the manuscript and have edited Figure 5 (previous Figure 7) accordingly.

Results, lines 389-395:

*“Comparable to the results of experiment 1 (****Figure 2Bii****), we observed differences between the stimulation conditions in the power at Fz, which again predominantly presented as differences in power within the alpha band, and primarily saw a greater power at around 8 Hz in pre-trough stimulation (****Figure 5A****). We focussed on power during the first 5 minutes of stimulation here, during which the majority of participants remained awake as in experiment 1 (the analysis including the full duration of the nap opportunity can be seen in* ***Figure S12****).”*

**3.** I was wondering how alpha stimulation was realized during sleep onset / light sleep. As explained by the authors falling asleep is characterized by a dramatic reduction in alpha activity. If alpha dissolves how is it possible to apply a closed loop procedure in a meaningful way (even more so targeting frontal sites were alpha is less dominant in general)?

This is again a very insightful point and one that we acknowledge as a limitation of our closed-loop algorithm. Because of this we wondered whether the ISIs of the sound stimulation would deviate away from alpha as the brain changed its state and consequently the predominant frequencies (i.e., alpha during wakefulness, theta during N1 and N2, sigma during N2). We noted that the ISIs shifted with brain state, and this happened even when the sound output was ‘off’ as in the case of the sham condition, where the algorithm was phased-locked to the pre-peak condition but there was no sound output, indicating that the closed-loop algorithm followed brain state (Figure S14). Nevertheless, we observed that a genuine alpha oscillation does not seem to be a requisite to induce changes in alpha frequency, as demonstrated in Figure 5B. Why exactly this is the case is not something we can conclude based on our current findings but might be perhaps explored using computational modelling.

We have expanded our Discussion to include the study specific relevant points presented above, lines 559-572:

*“A general limitation of our studies is that our closed-loop stimulation did not account for variations in the endogenous alpha power. This was mitigated by the fact that participants had their eyes closed, a state of abundant alpha oscillations, and variations in alpha power were used to our advantage in experiments 3-4. However, in experiment 5, we see that ISIs deviate to other frequencies when alpha oscillations are reduced as participants fall asleep. While we cannot completely rule out an effect of ISI, or stimulation frequency in experiment 5, such that the impact of sound on sleep is only phase-dependent in the sense that the frequency of sound was phase-dependent, the differences in ISI (0.4 Hz on average) are very slight, rendering this explanation unlikely. Future studies can implement closed-loop strategies that include a detection threshold, thus limiting stimulation to periods when the target rhythm is most prominent. Albeit the presence of a genuine alpha oscillation does not seem to be a requisite to induce changes in alpha frequency, as observed in experiment 5 where alpha oscillations are naturally reduced as participants fell asleep.”*

**4.** The result that participants had more trouble reaching stable sleep in the pre-trough condition is quite interesting. The authors suggest that this might be due to pre-trough αCLAS having impeded the disappearance of alpha (through increasing alpha power). Please show that pre-trough αCLAS indeed impacts alpha power during N1.

We had speculated that this might have been one of the possible mechanisms by which the pre-trough condition might have resulted in shallower sleep. But we appreciate that we have not provided evidence that this a phenomenon specific to N1 sleep.

Instead of focusing our analysis of sleep microstructure on specific sleep stages, we characterised both rhythmic, i.e. oscillatory, and arrhythmic, i.e. aperiodic or 1/f, EEG activity using eBOSC on the data obtained throughout the nap opportunity. We believe this approach offers more granularity with the advantage of not assuming that the brain just conforms to discrete states. Using this approach we have indeed provided evidence that features which decrease with sleep were greater during the pre-trough condition, i.e., exponent of 1/f slope (**Figure 6K**) and alpha abundance (representative of rhythmic alpha activity, **Figure 6H**).

Accordingly, we have now removed specific references to N1 and instead focused on alpha power and abundance of alpha oscillations generally, for which we have provided evidence, lines 545-552:

*“In experiments 1 and 5 we showed that pre-peak αCLAS predominantly caused a decrease in alpha power, whilst pre-trough αCLAS did the opposite, i.e. an increase in alpha power. Similarly, in experiment 5, we show that the abundance of alpha oscillations was greater in pre-trough stimulation than in either pre-peak or sham stimulation, despite these conditions not differing in their quantity of wake epochs. Accordingly, we suggest pre-trough αCLAS may have impeded the disappearance of alpha, preventing sleep from progressing further, whilst pre-peak αCLAS did not.”*

**Reviewer #3:**

Please note that we have divided the comments in points and have numerated those to better organise the structure of our responses.

**1.** In the present manuscript, Hebron et al,. introduce a novel approach to selectively modulate alpha oscillations using close loop auditory stimulation. First, I want to apologize for the late review. Unfortunately, this was the direct result of the fact that the manuscript is very dense and especially the beginning of the results section is difficult to follow and lacks important information to understand the approach. It took me multiple attempts to understand their approach and required jumping back and forth between intro, results and methods plus reading ref. 34 in order to understand what was actually done.

While the manuscript is potentially within the scope of PLoS Biology, it will require substantial revision to make it accessible for a broader audience.

We thank the reviewer for their willingness to understand our findings despite the density of the manuscript. We agree that we have presented many findings, and that the quantity might have distracted from the main message. We have now streamlined this version of the manuscript (also per suggestions from reviewer 1) to improve readability. We hope the current version is easier to follow and believe that the reviewer’s critique has contributed to an improved paper. As the reviewer will see, we have conducted a substantial revision, which includes new data and analyses.

We have included more information in the Results section to guide the reader through the analyses performed in each section.

In regard to the ecHT approach used (previous ref. 34) we have now added additional information in the Introduction to help the reader further understand the approach without having to fully revert to that paper.

Introduction, lines 49-51:

*“However, many time-frequency approaches provide phase estimates dependent on what proceeds them (i.e. they are non-causal), which might drive spurious correlations* [35]*.”*

Introduction, lines 78-80:

*“The ecHT is a method that tracks the instantaneous phase and envelope amplitude of an oscillatory signal, allowing the instantaneous phase to be reliably computed in real-time* [35,36]*.”*

**2.** The topic is straight-forward: How can we modulate oscillations in a spatially- and frequency-specific dependent manner? Previous studies attempted electrical or magnetic stimulation, resulting mostly in mixed results. Likewise, previous attempts using sensory stimulation remained equivocal. Here the authors introduce a new approach.

Unfortunately, this is where the manuscript falls short. First, there is no rationale why their approach should target alpha in a spatially selective manner. It seems crucial information from ref. 34 is missing in this manuscript in order to understand rationale and approach.

We absolutely agree with the reviewer regarding their summary of our findings and their novelty and importance. However, this summary can only be achieved *a posteriori*, i.e., once the data was analysed and the findings emerged. We had no previous grounds in which to hypothesise that we would be able to modulate alpha oscillations in a spatially- and frequency-specific manner, because this was never, to our knowledge, investigated in this manner.

**3.** On the contrary, the manuscript jumps right into data analysis without providing important sanity checks. What if the signal did not contain any oscillations to begin with? Then the filter would render the signal sinusoidal and provide the wrong impression that there was an oscillation in the beginning.

There are two important considerations to be addressed here. The first pertains to sanity checks, i.e., whether alpha oscillations were indeed present to be targeted by our αCLAS approach. The second relates to what would happen in the absence of those.

Regarding a sanity check for the presence of alpha oscillations. Please note that this was indeed our first analysis and was included in the manuscript and Figure 1. The analysis shows the expected presence of a spectral peak in the alpha band at both targeted electrodes (i.e., Fz and Pz) as would be expected given that participants were at rest with their eyes closed.

Results, lines 115-119:

*“The presence of a spectral peak in the alpha band (7.5-12.5 Hz) during the ‘off’ period was first confirmed in the phase-locking electrode from the αCLAS EEG system. A peak was observed in both the group average and majority of participants (experiment 1: 21/23; experiment 2: 28/28 participants,* ***Figure 1C****), demonstrating that alpha activity was present at the target electrode.”*

Regarding the second point, i.e., the fact that the filter would render the signal sinusoidal and deliver sound stimulation even without a true alpha oscillation. This is an important point, which we have fully acknowledged as a limitation of our closed-loop algorithm. This is particularly important in experiment 5 (previous study 3, i.e., nap study) where participants are falling asleep and alpha power will naturally become reduced. Because of this we wondered whether the ISIs of the sound stimulation would deviate away from alpha as the brain changed its state and consequently the predominant frequencies (i.e., alpha during wakefulness, theta during N1 and N2, sigma during N2). We noted that the ISIs shifted with brain state, and this happened even when the sound output was ‘off’ as in the case of the sham condition, where the algorithm was phased-locked to the pre-peak condition but there was no sound output, indicating that the closed-loop algorithm followed brain state (Figure S14). Nevertheless, we observed that a genuine alpha oscillation does not seem to be a requisite to induce changes in alpha frequency, as demonstrated in Figure 5B. Why exactly this is the case is not something we can conclude based on our current findings but might be perhaps explored using computational modelling.

We have expanded our Discussion to include the study specific relevant points presented above, lines 559-572:

*“A general limitation of our studies is that our closed-loop stimulation did not account for variations in the endogenous alpha power. This was mitigated by the fact that participants had their eyes closed, a state of abundant alpha oscillations, and variations in alpha power were used to our advantage in experiments 3-4. However, in experiment 5, we see that ISIs deviate to other frequencies when alpha oscillations are reduced as participants fall asleep. While we cannot completely rule out an effect of ISI, or stimulation frequency in experiment 5, such that the impact of sound on sleep is only phase-dependent in the sense that the frequency of sound was phase-dependent, the differences in ISI (0.4 Hz on average) are very slight, rendering this explanation unlikely. Future studies can implement closed-loop strategies that include a detection threshold, thus limiting stimulation to periods when the target rhythm is most prominent. Albeit the presence of a genuine alpha oscillation does not seem to be a requisite to induce changes in alpha frequency, as observed in experiment 5 where alpha oscillations are naturally reduced as participants fell asleep.”*

**4.** Likewise, it is puzzling how their systems can be placed at electrodes Pz/Fz, but simultaneous HD-EEG is recorded at exactly the same locations.

We apologise for our lack of clarity regarding the ‘same’ location for the electrodes. We have now clarified this indicating that the Fz and Pz electrodes in the hd-EEG system were in adjacent positions to those in the αCLAS EEG system.

Introduction, lines 132-134:

*“We then measured the resultants for the equivalent electrodes in the hd-EEG system (Fz and Pz in the hd-EEG were adjacent to those in the αCLAS EEG system).”*

Methods, lines 702-703:

*“The hd-EEG electrodes matching those used for phase-locking in the αCLAS EEG system (i.e., Fz or Pz) were moved to be adjacent to those in the αCLAS EEG system.”*

**5.** Second, the employed null model is far from optimal. Their null model predicts random phase - this however, is not correct. The null model is that there is no oscillation, but that all effects can be explained by evoked responses. Seminal work by e.g., Klimesch and colleagues over a decade ago already showed that evoked responses are modulated in a frequency- and phase-specific manner, hence, are not independent of ongoing activity (no additive model). The present results can be interpreted in the same vein. Recent work on the lack of entrainment or alternative approaches should be considered as their null model: e.g., Oganian 2020 Sci Adv or Oganian 2023 JNEurosci for comparable approach in MEG or Breska and Deouell 2017 or Obleser 2017 PLoS Biol. For related discussion see e.g. Haegens 2020 or Helfrich, Breska et al. 2019.

We believe that these models make sense for interrogating how the brain’s activity changes with speech; an aperiodic stimulus which varies in its rate. In this literature, it is often common to contrast speech at different rates, predicting that oscillatory entrainment will see consistent changes at a given frequency (e.g., theta oscillations will track speech of varying rates) whilst in the case of an evoked potential the changes will occur at, or close to, the frequency of the speech.

However, it must be recognised that these papers (e.g., Oganian 2020) do not provide a compelling argument either way, the data suggest a mixture of models. It is possible, of course, that oscillations and evoked potentials are not mutually exclusive in time and space, and that a stimulus can evoke a potential and entrain an oscillation. Indeed, the idea that neural activity tracks the envelop of speech is not without debate. Currently, two main competing models have been put forward: entrainment (i.e., phase alignment) and transient neural responses [19–23]. However, the jury is still out to whether entrainment is present when speech is not rhythmic. Still, the models use to evaluate the likelihood of entrainment vs evoked potential would not apply to our study since we do not have different stimuli at very different frequencies.

Moreover, entrainment is typically defined as synchronization in phase or amplitude of neural oscillatory activity to periodic stimulation. Based on this definition, stimulus periodicity is a requisite. To test whether our closed-loop approach delivered stimuli in a periodic fashion we tested the autocorrelation of the inter stimulus interval across 20 lags, in which a value of 1 would indicate a perfectly periodic stimulus and lower values indicate a deviation from periodicity (see figure below). The 4 different colours in the figure represent the four phase conditions and the error bars indicate standard error of the mean with ISIs retrieved from experiment 1, which contains blocks of repetitive stimulation. It is clear that the stimulation we administered is far from periodic, with the autocorrelation coefficient quickly dropping to < 0.1 after only 2 lags. We propose that if the sound was simply entraining itself, via the EEG, then we would see a highly periodic stimulus. This is not the case here (see Figure below, which has now been added as Figure S6).

We have now included information in the manuscript and supplementary files according to the analyses we presented above.

Results, lines 225-227

*“The effects of αCLAS on power and frequency cannot be simply explained by entrainment to a periodic stimulus, as the stimuli delivered by αCLAS was aperiodic (****Figure S6****).”*

In relation to the additive vs phase-reset and other models used to explain the origin of evoked potentials. This is a topic that was not the focus of our investigation and one that continues to be debated in the literature [2–16]. Current evidence suggests that the differentiation between these models might not be feasible if based solely on macroscopic EEG recordings, indicating that invasive recordings are needed to disentangle what model is most appropriate [4,17].

We have now included additional information on the current literature discussion on the difficulty to adjudicate between models of the origins of evoked potentials.

Discussion, lines 514-519

*“There is no unanimous consensus on how related or unrelated evoked potentials are to phase reset of oscillations* [24] *with ongoing attempts to adjudicate between the best models to explain the generation of evoked responses from EEG data* [2–16]. *Current evidence suggests that the differentiation between these models might not be feasible if based solely on non-invasive scalp EEG recordings* [4,17]*”*

Furthermore, the closed-loop nature of our experiments makes creating a null-model of phase based on our data unfeasible. The closed-loop is designed to ensure that the frequency of the EEG and the stimulus are tightly linked.

Finally, other studies mentioned by the reviewer are hard to compare with our current study. Those studies utilise a task paradigm with specific attentional demands or attention to speech, which require higher order processing and integration of information. Our study used pink noise, participants were at rest, with their eyes closed and no task to be performed. In future studies others might want to explore the application of closed-loop paradigms in a task to assess cognitive functions such as attention.

**6.** While the manuscript considers different possibilities in Fig. 5, this part needs to appear much earlier and dissect whether we are facing the result of evoked responses or genuine phase entrainment.

We thank the reviewer for this critical question on precisely what is evoked by the sounds as employed here. The distinction between the two cases (evoked potentials vs phase-reset of oscillations) is exceptionally difficult since both cases are likely to present in the same manner in the EEG. Also see response to the previous question regarding this debate.

Whatever the source of the effect (genuine phase-reset of oscillations or simple addition/superposition of potentials), it is temporally coupled to ongoing alpha band activity in the case of alpha phase-locked stimulation. This means it will appear to be part of alpha activity, whether it shares an origin or not. This is especially problematic in a measure like EEG, in which electrophysiological features can have independent sources and also appear superimposed in space at the scalp, making them impossible to fully separate. This issue is compacted further by the notion of ‘cortical excitability’, which supposes that the brain will be more or less excitable, depending on the phase of ongoing oscillations. If such a principle exists for alpha oscillations and sounds, in addition to an additive model, separation becomes yet more difficult. For example, if the brain is most excitable at the peak of an oscillation, then in simple terms a larger evoked potential may be elicited at this phase. However, the positive deflection of that potential may be perfectly aligned with the trough of the ongoing alpha, making it appear smaller in magnitude through some process of cancelling out. In the opposite example, the trough is the least excitable but the potential is added to the already positive peak of a subsequent oscillation.

In the case of a pure phase-reset this would not be the case, assuming that every and all oscillators contributing to alpha activity are reset in precisely the same manner. This seems unlikely, since we measure innumerable oscillators from may different sources, and may not reset all of them. This leaves us with a situation in which some oscillators are reset and some continue, another situation in which an additive and phase-reset model may not be distinguished in the EEG.

Despite these difficulties in disentangling oscillations from evoked potential in a closed-loop, such as ours, we have made our best attempt to describe below what might be occurring. However, it must be said, it remains somewhat ambiguous.

In order to show the immediate response in the EEG to repetitive aCLAS we plotted ERP’s for each of the first 10 stimuli in experiment 1 where we used a block design with repetitive αCLAS. Below is shown, for each stimulus, the evoked potential at Fz for each phase condition (coloured lines), the average of all four conditions (black line), the stimulus onset (vertical grey line) and statistically significant differences from zero across all subjects and conditions (red scatter points, significantly greater than zero, blue scatter points, significantly less than zero; EEG data filtered between 1-40 Hz as in Figure 3 Ai). What is clearly shown is that the first stimulus evokes a, broadly, conventional response to a sound, with high frequency components followed by a low frequency component. This is especially clear when averaging across conditions, and it is also evident that the low frequency component of this evoked potential moves steadily closer to stimulus onset with each subsequent stimulus, demonstrating that it is only the first stimulus which evokes this low frequency response. Note: the frequency of stimulation is according to the alpha frequency, i.e., there is a stimulus every 100 ms or so, and these plots are describing around 1 second of stimulation. Once this low-frequency component has passed the predominant response in the EEG is a short-lived higher frequency component. As always, this component may just as convincingly represent either a superimposed evoked potential or a short-lived phase rest of non-stationary oscillations.

The specificity of this low frequency potential to the first stimulus can be seen yet more clearly when filtering the EEG using a 5 Hz lowpass filter, see below:

For consistency with our other analyses and considering our interest in the alpha band in particular, we also plotted the same data filtered 7.5-12.5 Hz. As with previous analyses, it can be seen that conditions are orthogonal before a stimulus and more aligned after a stimulus. An oscillation post-stimulus, greater in magnitude than pre-stimulus, can be seen in both the average trace (black line) and the timepoints at which the group was greater or less than zero (red and blue scatter points, respectively).

This effect can be seen more clearly still, when applying a Hilbert transform to the alpha-filtered signal to obtain phase (coloured lines) and conducting a Rayleigh test to identify periods of statistically significant phase homogeneity between conditions (black scatter points):

Whilst these analyses are consistent with our phase-reset model of how the stimulation may be affecting the EEG, they do not entirely resolve the issue of phase-resets vs evoked-potentials. We can clearly see that a large evoked potential, as seen following the first stimulus, cannot reasonably be entirely responsible for the observed effect, but we cannot comment definitively on the nature of the higher frequency component evoked by subsequent stimuli.

One simple way in which these things have been explored before is to investigate whether there is a change in power in the band of interest (i.e., that which you suspect is being reset – in our case, alpha), the rationale being that an additive (or evoked potential model) effect would increase power in the band of interest (i.e., it would *add* to it), whilst a genuine phase reset would not [4].When conducting an analysis of this sort (again taking data from experiment 1), we do not see a change in the total alpha bandpower at our frontal ROI in experiment 1. Below **A** shows the topography of the ANOVA conducted on total alpha power (7.5-12.5 Hz) – no main effect of stimulus phase is observed. **B** shows the changes in alpha power (7.5-12.5 Hz) across conditions at the frontal ROI in experiment 1. In none of our conditions do we see a significant increase in alpha power, nor do we observe an effect of stimulus phase on total alpha power. This, we propose, is because the power in the alpha band is simply being redistributed by stimulation (i.e., a frequency change, i.e., a phase-reset).

Another approach taken [4] to distinguish an additive from a phase-reset mechanism is to compute the evoked power. That is the power spectrum of the averaged ERP. The rationale being that a phase-reset will align trials and hence an oscillation will emerge in the average, the frequency of which may tell you which oscillations are being reset. Below is shown **A** the evoked potential at Fz (again from experiment 1), averaged across participants and conditions, with red marks indicating significantly higher than zero and blue marks indicating significantly lower and vertical lines indicating intervals of 100 ms. The evoked potential appears to contain a ~10 Hz oscillation. Also show is **B** the change in evoked power from pre to post stimulus, also showing an increase at 10 Hz with significant differences indicated by black marks. Now, this analysis alone, in our case, presents a problem because of the repetitive nature of our stimulus – one could suggest entrainment. But, paired with the somewhat aperiodic nature of our stimulus (see plot in previous response) and the fact that we did not see an increase in alpha power (see above) we are inclined to maintain our phase-reset mechanism.

One of the other criteria used in the same investigation of phase-reset vs additive mechanisms [4] is the phase distribution. The authors suggest that a phase reset would result in a non-uniform phase distribution in the band of interest, since the oscillations are being reset. We have shown the same phenomenon before in the manuscript, and again above in our response.

Regardless, it is important for us to highlight that (despite our willingness to engage with this popular debate on phase-resets, entrainment, evoked potentials, etc) the broad purpose of our investigation, and this manuscript, is to employ a new technology in an exploratory fashion and to observe its effects on the brain. We believe the phase-reset model is sufficient to describe the origin of these observations, what this precisely means for the brain is a separate matter. At any rate, it is not immediately obvious to what extent, and on what scale, the origin of a field potential (evoked, entrained, oscillation, and so on) actually matters for brain function – one would imagine the individual neuron doesn’t very much mind why its firing for example. If we consider the brain as a network of oscillators, on the other hand, then the differences between these various proposed mechanisms may become more significant, but where the line between these two scales becomes meaningful is difficult to assess.

**7.** Likewise, the results from the connectivity analysis are meaningless if we solely face evoked responses. Either way, sensor level connectivity is not a great way to assess functional interactions and it is preferable to conduct in source space using metrics that correct for volume spread (e.g., Hipp 2011 Neuron or 2012 Nat Neuro).

While we appreciate the conviction that evoked potentials hold no meaning for communication between different areas of the brain, we would prefer to be slightly more cautious. For example, it is not straightforward for us to imagine an evoked potential which can increase or decrease connectivity (with different topographies mind you) purely on the basis of the phase (at Fz) at which it is administered. If opposite phase conditions offered opposite effects on the connectivity, then we may be more inclined to dismiss them outright, but this is not what we observe.

Regarding the question of sensor vs source. The primary objective with source space analysis is to make more accurate neuroanatomical inferences from M/EEG signal. This was not the objective of our connectivity analyses, where we ‘simply’ wanted to investigate whether an effect of phase on connectivity was present and the general pattern this effect might have.

We appreciate that connectivity at the sensor level comes with a set of assumptions, but would argue that source reconstruction adds yet more assumptions. Source reconstruction requires complex pipelines broadly divided into addressing forward and inverse problems. The forward problem is the easiest to solve, provided that one has realistic and subject-specific head models obtained from MRI data. As part of this study, we did not acquire MRI scans from our participants and therefore cannot build individual head models. The inverse problem is then addressed by generating scalp distributions from electrical potential differences based on a specific algorithm. There are, however, a plethora of inversion algorithms available, persistent controversies regarding the choice of algorithm, with many studies suggesting that more than one algorithm should be compared to establish the reliability of the results (e.g., [31,32]). We also consider that our closed-loop approach may present some unique obstacles for source reconstruction, since it is enforcing a strong correlation between the auditory-evoked activity and the spontaneous activity, and source reconstruction seeks to separate sources on the basis of their correlation.

A recent study comparing functional connectivity estimates from sensor and source spaces concluded that there is a strong correlation for the global connectivity between scalp- and source-level [31]. In contrast, network topology (not investigated in our study) was only weakly correlated, albeit more accurate for functional connectivity metrics that limit the effects of volume conduction/signal leakage, such as PLI. This is also why we employed both PLV and PLI in our analysis of connectivity.

In order to reduce the number of results presented in the main manuscript and considering that the connectivity analysis is not our main focus, we have now moved it to the supplementary materials. We believe this will aid in focusing our narrative on the main findings presented in the manuscript.

**8.** Moreover, given the 3 included studies, the manuscript is very dense and fails to specify details in numerous instances. For example, in Fig. 1 it remains unclear why activity as estimated from Fz differs between ecHT or HD-EEG, it must surely be the same position? The authors point towards different referencing schemes, but do not address how these were placed simultaneously.

We thank the reviewer for highlighting this issue which we have now addressed.

Below is the phase-locking accuracy (resultant) at Fz (taken from the hd-EEG system) in experiment 1, contrasting the Laplacian reference with the right mastoid reference (taken from TP10 also in the hd-EEG system). There is a subplot for each phase condition. Statistics show the result of a paired t-test comparing the two referencing schemes, *** p <.001. The phase-locking accuracy is higher when referencing to the right mastoid, as expected considering the reference used for the ecHT device.

We have now added this information in the revised manuscript and supplementary material.

Introduction, lines 136-140:

*“The lower resultant vector lengths displayed for the hd-EEG are a consequence of the scalp current density (i.e., Laplacian) reference used here (see Figure S1 for a comparison between resultants at the target electrode in the hd-EEG system using Laplacian or mastoid reference as in the αCLAS EEG system).”*

***Figure S1. Comparison of the resultant values using different referencing schemes.*** *The resultant was calculated in electrode Fz of the hd-EEG system in Experiment 1 using two referencing schemes, Laplacian (as used for all analysis in the main manuscript) and right mastoid (to match the referencing scheme used in the αCLAS EEG system). Channel Fz in the hd-EEG system was adjacent to this channel in the αCLAS EEG system, and the mastoid channel in the hd-EEG system was taken from TP10. The resultant values using the right mastoid reference are higher and more similar to those reported for the αCLAS EEG system.*

*Violin plots show the resultant for each targeted phase in Experiment 1 in each reference scheme. Black lines represent each participant. Stats indicate output of paired samples t-test between the resultant for each reference scheme, ***p<0.001.*

**9. Likewise, the z-scoring procedure remains unclear, to what distribution did the authors z-score**? The tests for resultant vector length were not specified. It can be assumed these are Rayleigh z and not a typical z-scores?

There are three different Z-scores used depending on the requirements of the specific analysis in question. Firstly, the power spectra in Figure 1 are normalized by z-scoring (i.e., power at each frequency is expressed as number of standard deviations from the average power over all frequencies), secondly there are z-scores which result from V-tests for checking phase-locking accuracy in Figure 1 (i.e., comparing a phase distribution to a pre-specified phase angle, and arriving at statistical significance if they are sufficiently similar and sufficiently unimodal, akin to a directional Rayleigh test), and thirdly there are z-statistics resulting from Rayleigh tests in Figure 3 (in this case, we had no a priori expectation of phase angle following stimulation, and simply wanted to test for unimodality). We have made this clearer in both the figure legend and the methods section.

The Figure 1 legend now reads:

*“****(C)*** *Normalised (z-score;* ***i.e., power each frequency is expressed as SD’s from mean power across all frequencies, per participant****) power spectrum computed for ‘off’ periods, taken from the phase-locking (i.e. ecHT) electrode in* ***(i)*** *experiment 1 (Fz) and* ***(ii)*** *experiment 2 (Pz).”*

And

*“p-values from FDR-corrected* ***V-test for directionality****.”*

The Figure 3 legend now reads:

*“Pre-stimulus alpha power vs maximum post-stimulus Z-statistic (****resulting from Rayleigh test, and demonstrating significant unimodality****, i.e. phase synchrony across conditions)”*

And the methods section has been updated to read, lines 765-772:

*“Power spectra in Figure 1C were computed from the off-period using the αCLAS EEG system, and were normalised by z-scoring, i.e., expressing power at each frequency as number of standard deviations from the mean power, per participant. This was so as to plot all participants together, without individual differences leading to large disparities between participants’ power spectra. The actual power values here are of no consequence, since the aim was only to display a peak within the alpha band. Peaks were detected using the MATLAB function ‘findpeaks.m’. If a peak was identified within the alpha band used by the αCLAS system (i.e., 7.5-12.5 Hz) it was marked on the plot.”*

**10.** Why should sound stimulation be spatially selective and not engage posterior channels as well when frontal activity is targeted? Similar considerations apply to all other figures.

This question is exactly at the root of the reason we tested our αCLAS approach at two different locations. As we mentioned previously, we could not have known *a priori* that the effects would have been spatially specific. This makes this approach particularly powerful, as it will allow others to design experiments to target alpha at different cortical locations.

In addition, this question relates to question 15 posed by Reviewer 1: *“How far were the phases of Fz from those stimulated on Pz? What is the phase offset between these two scalp locations, and is it predictable or random?”*

Indeed, if the phase offset was predictable and consistently different in a particular phase targeting Fz might have specific effects at Pz. Below is the phase offset between Fz and Pz, at stimulus onset, following Laplacian reference, across all stimuli, across all 4 conditions, in experiment 1. Each line represents an individual participant, the angle represents the average phase offset, and the length of these lines represents the resultant. Whilst the majority of participants exhibit a roughly opposite phase between Fz and Pz, the resultant is quite low (<0.4 for most participants) and the spread is not especially focused. The low resultant indicates that the phase offset is not predictable and provides some explanation to the spatial specificity of the effects since the alpha phases at these two locations do not have a tight relationship.

**11.** Paragraph 2 then starts with 'Having established the accuracy and spatial-specificity of αCLAS..'. Here I think we are facing a major hurdle if the basics remain ill specified.
Since all subsequent results would differ depending on the outcome of the control analyses with regard to Figure 1, I would suggest that the authors first revise these essential points and then the remainder of the manuscript needs to be reassessed.  If we take the perspective that we are currently facing mainly the results of evoked responses, then the subsequent results need to be rephrased.

We hope that the answers given above and all the changes we made to the manuscript have provided further clarity.

We have also changed the sentence the reviewer mentioned to be more exact on our specific observations at that stage in the paper.

Introduction, lines 182-184:

*“Having shown that αCLAS delivers sounds accurately at the prescribed phase in the two targeted electrode locations, we next examined whether αCLAS induced phase-dependent changes to the alpha oscillations we set out to target.”*

**References**

1.	Nunez PL, Srinivasan R. Electric fields of the brain: the neurophysics of EEG. Oxford University Press, USA; 2006.

2.	Iemi L, Busch NA, Laudini A, Haegens S, Samaha J, Villringer A, et al. Multiple mechanisms link prestimulus neural oscillations to sensory responses. Hanslmayr S, Colgin L, editors. Elife. 2019;8: e43620. doi:10.7554/eLife.43620

3.	Sauseng P, Klimesch W, Gruber WR, Hanslmayr S, Freunberger R, Doppelmayr M. Are event-related potential components generated by phase resetting of brain oscillations? A critical discussion. Neuroscience. 2007;146: 1435–1444. doi:10.1016/j.neuroscience.2007.03.014

4.	Hanslmayr S, Klimesch W, Sauseng P, Gruber W, Doppelmayr M, Freunberger R, et al. Alpha Phase Reset Contributes to the Generation of ERPs. Cerebral Cortex. 2007;17: 1–8. doi:10.1093/cercor/bhj129

5.	Min B-K, Busch NA, Debener S, Kranczioch C, Hanslmayr S, Engel AK, et al. The best of both worlds: Phase-reset of human EEG alpha activity and additive power contribute to ERP generation. International Journal of Psychophysiology. 2007;65: 58–68. doi:https://doi.org/10.1016/j.ijpsycho.2007.03.002

6.	Fuentemilla Ll, Marco-Pallarés J, Grau C. Modulation of spectral power and of phase resetting of EEG contributes differentially to the generation of auditory event-related potentials. Neuroimage. 2006;30: 909–916. doi:https://doi.org/10.1016/j.neuroimage.2005.10.036

7.	Fell J, Dietl T, Grunwald T, Kurthen M, Klaver P, Trautner P, et al. Neural Bases of Cognitive ERPs: More than Phase Reset. J Cogn Neurosci. 2004;16: 1595–1604. doi:10.1162/0898929042568514

8.	Gruber WR, Klimesch W, Sauseng P, Doppelmayr M. Alpha Phase Synchronization Predicts P1 and N1 Latency and Amplitude Size. Cerebral Cortex. 2005;15: 371–377. doi:10.1093/cercor/bhh139

9.	Klimesch W, Schack B, Schabus M, Doppelmayr M, Gruber W, Sauseng P. Phase-locked alpha and theta oscillations generate the P1–N1 complex and are related to memory performance. Cognitive Brain Research. 2004;19: 302–316. doi:https://doi.org/10.1016/j.cogbrainres.2003.11.016

10.	Makeig S, Westerfield M, Jung T-P, Enghoff S, Townsend J, Courchesne E, et al. Dynamic Brain Sources of Visual Evoked Responses. Science (1979). 2002;295: 690–694. doi:10.1126/science.1066168

11.	SAVERS BMCA, BEAGLEY HA, HENSHALL WR. The Mechanism of Auditory Evoked EEG Responses. Nature. 1974;247: 481–483. doi:10.1038/247481a0

12.	Mazaheri A, Jensen O. Asymmetric Amplitude Modulations of Brain Oscillations Generate Slow Evoked Responses. The Journal of Neuroscience. 2008;28: 7781. doi:10.1523/JNEUROSCI.1631-08.2008

13.	Nikulin V V, Linkenkaer-Hansen K, Nolte G, Lemm S, Müller KR, Ilmoniemi RJ, et al. A novel mechanism for evoked responses in the human brain. European Journal of Neuroscience. 2007;25: 3146–3154. doi:https://doi.org/10.1111/j.1460-9568.2007.05553.x

14.	Mazaheri A, Jensen O. Posterior α activity is not phase-reset by visual stimuli. Proceedings of the National Academy of Sciences. 2006;103: 2948–2952. doi:10.1073/pnas.0505785103

15.	Mäkinen V, Tiitinen H, May P. Auditory event-related responses are generated independently of ongoing brain activity. Neuroimage. 2005;24: 961–968. doi:https://doi.org/10.1016/j.neuroimage.2004.10.020

16.	Shah AS, Bressler SL, Knuth KH, Ding M, Mehta AD, Ulbert I, et al. Neural Dynamics and the Fundamental Mechanisms of Event-related Brain Potentials. Cerebral Cortex. 2004;14: 476–483. doi:10.1093/cercor/bhh009

17.	Telenczuk B, Nikulin V V, Curio G. Role of Neuronal Synchrony in the Generation of Evoked EEG/MEG Responses. J Neurophysiol. 2010;104: 3557–3567. doi:10.1152/jn.00138.2010

18.	Alexandrou AM, Saarinen T, Kujala J, Salmelin R. Cortical entrainment: what we can learn from studying naturalistic speech perception. Lang Cogn Neurosci. 2020;35: 681–693. doi:10.1080/23273798.2018.1518534

19.	Capilla A, Pazo-Alvarez P, Darriba A, Campo P, Gross J. Steady-State Visual Evoked Potentials Can Be Explained by Temporal Superposition of Transient Event-Related Responses. PLoS One. 2011;6: e14543-. Available: https://doi.org/10.1371/journal.pone.0014543

20.	Giraud A-L, Poeppel D. Cortical oscillations and speech processing: emerging computational principles and operations. Nat Neurosci. 2012;15: 511–517. doi:10.1038/nn.3063

21.	Ding N, Melloni L, Zhang H, Tian X, Poeppel D. Cortical tracking of hierarchical linguistic structures in connected speech. Nat Neurosci. 2016;19: 158–164. doi:10.1038/nn.4186

22.	Breska A, Deouell LY. Neural mechanisms of rhythm-based temporal prediction: Delta phase-locking reflects temporal predictability but not rhythmic entrainment. PLoS Biol. 2017;15: e2001665-. Available: https://doi.org/10.1371/journal.pbio.2001665

23.	Zoefel B. Speech Entrainment: Rhythmic Predictions Carried by Neural Oscillations. Current Biology. 2018;28: R1102–R1104. doi:https://doi.org/10.1016/j.cub.2018.07.048

24.	Sauseng P, Klimesch W, Gruber WR, Hanslmayr S, Freunberger R, Doppelmayr M. Are event-related potential components generated by phase resetting of brain oscillations? A critical discussion. Neuroscience. 2007;146: 1435–1444. doi:https://doi.org/10.1016/j.neuroscience.2007.03.014

25.	Dijk D-J, Groeger JA, Stanley N, Deacon S. Age-Related Reduction in Daytime Sleep Propensity and Nocturnal Slow Wave Sleep. Sleep. 2010;33: 211–223. doi:10.1093/sleep/33.2.211

26.	Berry RB, Brooks R, Gamaldo C, Harding SM, Lloyd RM, Quan SF, et al. AASM Scoring Manual Updates for 2017 (Version 2.4). Journal of Clinical Sleep Medicine. 2024;13: 665–666. doi:10.5664/jcsm.6576

27.	Kosciessa JQ, Grandy TH, Garrett DD, Werkle-Bergner M. Single-trial characterization of neural rhythms: Potential and challenges. Neuroimage. 2020;206: 116331. doi:https://doi.org/10.1016/j.neuroimage.2019.116331

28.	Fehér KD, Wunderlin M, Maier JG, Hertenstein E, Schneider CL, Mikutta C, et al. Shaping the slow waves of sleep: A systematic and integrative review of sleep slow wave modulation in humans using non-invasive brain stimulation. Sleep Med Rev. 2021;58: 101438. doi:https://doi.org/10.1016/j.smrv.2021.101438

29.	Jasiukaitis P, Hakerem G. The Effect of Prestimulus Alpha Activity on the P300. Psychophysiology. 1988;25: 157–165. doi:https://doi.org/10.1111/j.1469-8986.1988.tb00979.x

30.	Brandt ME, Jansen BH. The Relationship Between Prestimulus Alpha Amplitude and Visual Evoked Potential Amplitude. International Journal of Neuroscience. 1991;61: 261–268. doi:10.3109/00207459108990744

31.	Lai M, Demuru M, Hillebrand A, Fraschini M. A comparison between scalp- and source-reconstructed EEG networks. Sci Rep. 2018;8: 12269. doi:10.1038/s41598-018-30869-w

32.	Mahjoory K, Nikulin V V, Botrel L, Linkenkaer-Hansen K, Fato MM, Haufe S. Consistency of EEG source localization and connectivity estimates. Neuroimage. 2017;152: 590–601. doi:https://doi.org/10.1016/j.neuroimage.2017.02.076

33.	Klimesch W, Sauseng P, Hanslmayr S. EEG alpha oscillations: The inhibition–timing hypothesis. Brain Res Rev. 2007;53: 63–88. doi:https://doi.org/10.1016/j.brainresrev.2006.06.003

34.	Schalk G. A general framework for dynamic cortical function: the function-through-biased-oscillations (FBO) hypothesis. Front Hum Neurosci. 2015;9: 352. doi:10.3389/fnhum.2015.00352

35.	Vinao-Carl M, Gal-Shohet Y, Rhodes E, Li J, Hampshire A, Sharp D, et al. Just a phase? Causal probing reveals spurious phasic dependence of sustained attention. Neuroimage. 2024;285: 120477. doi:https://doi.org/10.1016/j.neuroimage.2023.120477

36.	Schreglmann SR, Wang D, Peach RL, Li J, Zhang X, Latorre A, et al. Non-invasive suppression of essential tremor via phase-locked disruption of its temporal coherence. Nat Commun. 2021;12: 363. doi:10.1038/s41467-020-20581-7
